# Interface Engineering Strategies for Shuttle Mitigation in Alkali Metal–Sulfur Batteries: A Comparative Review from Li–S to Na–S and K–S Systems

**DOI:** 10.1007/s40820-025-02004-8

**Published:** 2026-01-05

**Authors:** Zihan Chen, Qiyao Yu, Wei Wang, Jianguo Zhang

**Affiliations:** 1 School of Mechatronical Engineering, Beijing Institute of Technology, Beijing, 100081 People’s Republic of China; 2 School of Metallurgical and Ecological Engineering, University of Science and Technology, Beijing, Beijing, 100083 People’s Republic of China

**Keywords:** Alkali metal–sulfur battery, Interface engineering, Shuttle mitigation, Common and inherent differences

## Abstract

Rechargeable alkali metal-sulfur (M–S) batteries, including Li/Na/K–S chemistries, have the potential to utilize abundant and low-cost sulfur cathodes yet offer high theoretical energy densities. However, their practical electrochemical performance is fundamentally limited by the polysulfide shuttle effect. This challenge is particularly exacerbated in Na–S and K–S systems owing to larger metal-ion radii, weaker solvation energies, slower redox kinetics, and greater electrolyte–electrode incompatibilities compared to Li–S batteries. This review presents a comparative analysis of interface engineering strategies designed to suppress the shuttle effect across these three systems. Following a summary of sulfur cathode properties and reaction mechanisms, we systematically examine the origins of polysulfide shuttling. Our analysis progresses from functional separator design and interlayer enhancements to the implementation of solid‑state electrolytes for root-cause inhibition. By evaluating interface engineering research specific to Na–S and K–S batteries, we elucidate both shared principles and unique challenges inherent to alkali M-S systems. Finally, we propose multifaceted solutions to achieve shuttle-free operation and enhance overall battery performance, thereby establishing a foundation for future advancements.

## Highlights

- The inherent differences and connections in interface engineering for inhibiting shuttle effects in alkali M (Li, Na, K)-S batteries are reviewed.
- The research progress on the application of internal interface engineering in shuttle suppression is summarized.
- The shuttle effect challenge analysis of Li-, Na- and K-S batteries and the prospect of the development direction of the next generation of alkaline M-S batteries are proposed.

## Graphical abstract

**Figure:**
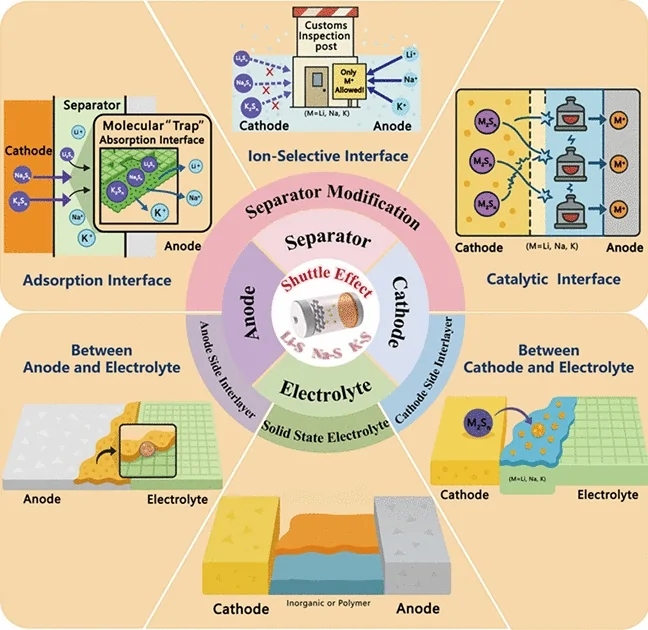

## Introduction

**Fig. 1:**
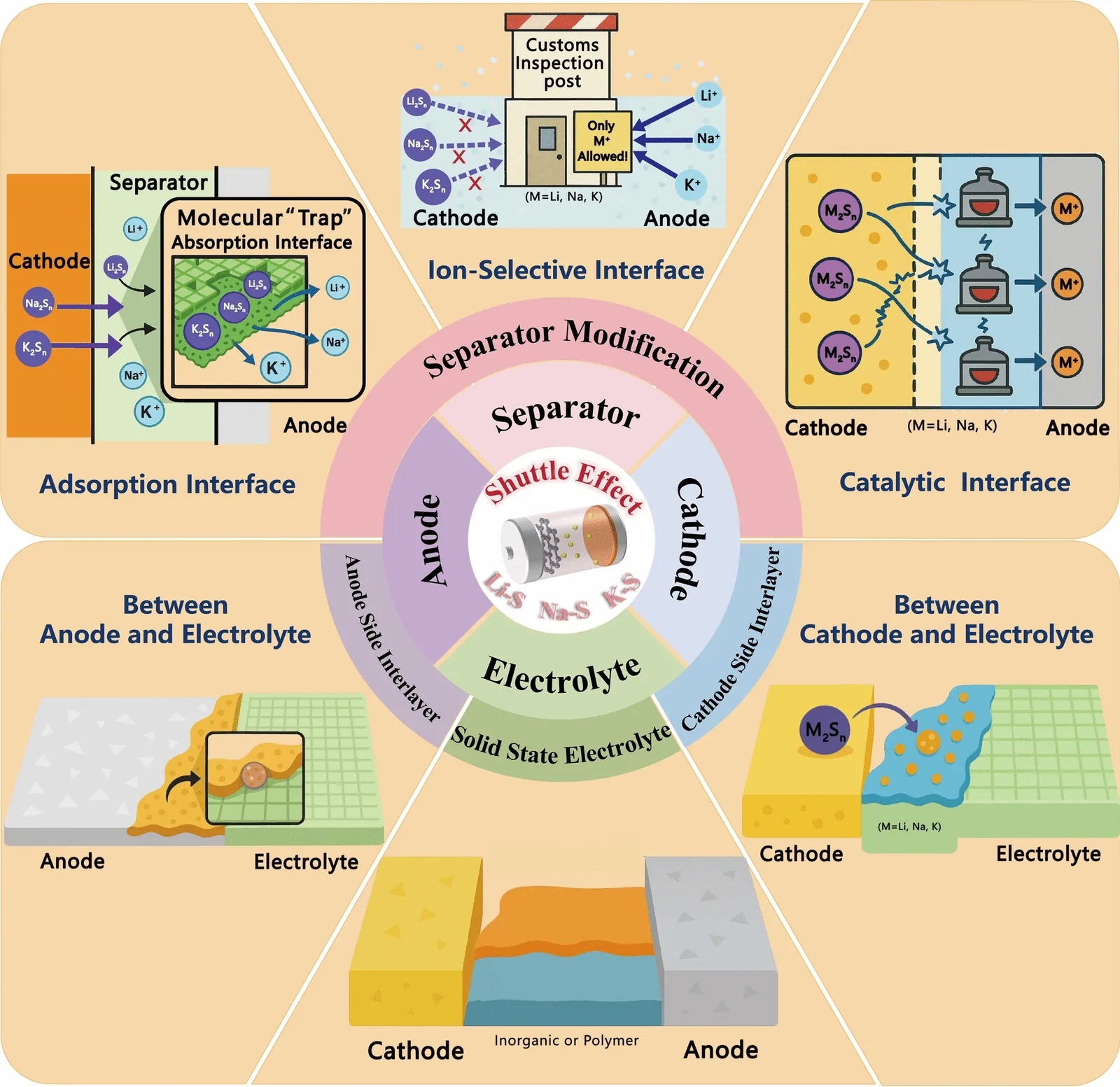
Schematic diagram of interface engineering strategies for inhibiting shuttle effects in M-S batteries

**Fig. 2:**
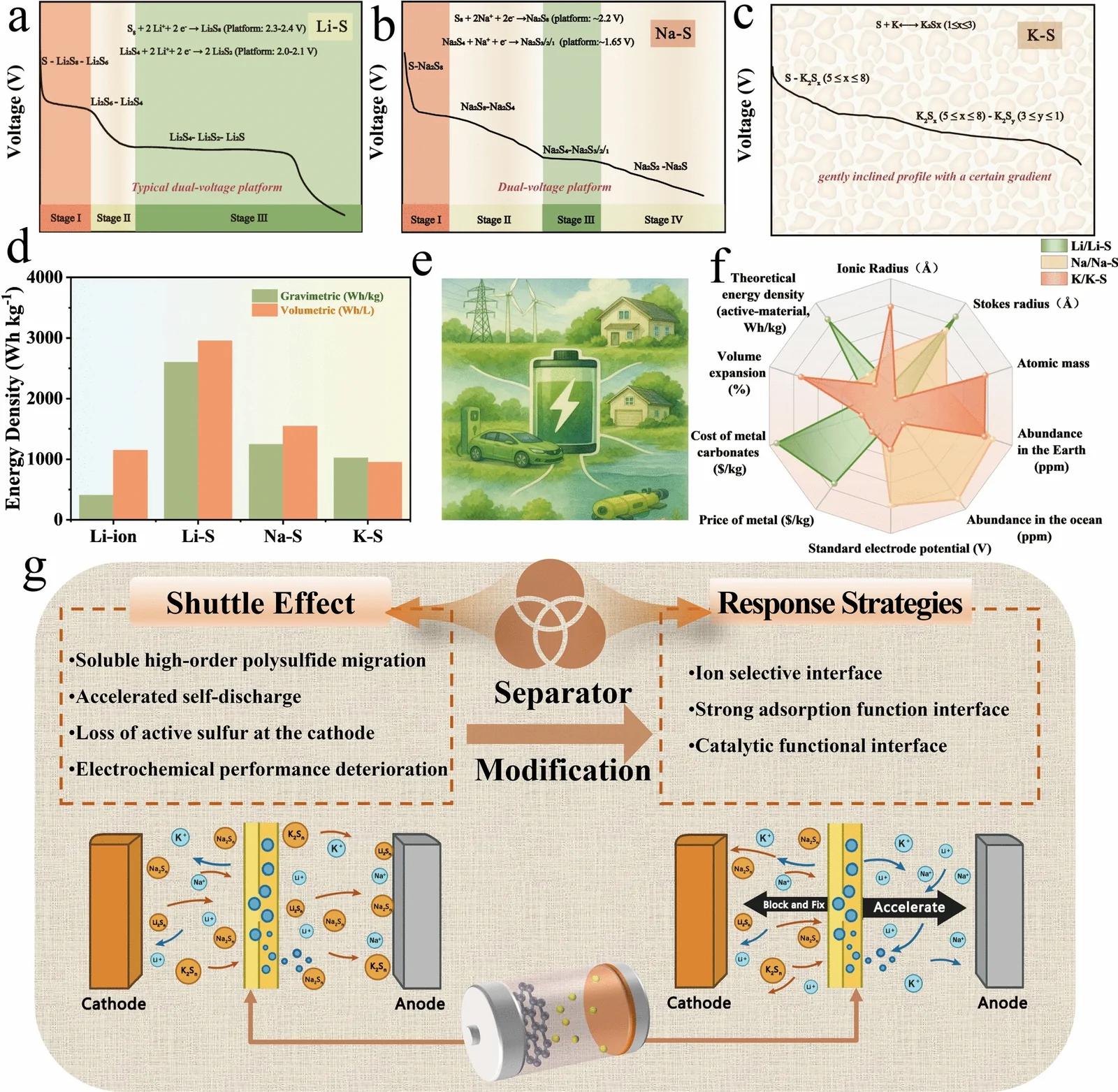
Typical discharge voltage profile of **a** Li–S cell. **b** Na–S cell with ether-based electrolyte. **c** K–S cell. **d** Comparison of the theoretical energy density of different batteries. (The AIBs values correspond to practical cell-level energy densities, which include the cathode, anode, separator, electrolyte, and current collectors, but exclude housings. For M-S batteries, the plotted values represent the theoretical active-material energy densities, calculated based on the specific capacity of sulfur and the average discharge voltage.) **e** Sustainable ecological network for battery energy storage and application. **f** Comparison of physical and chemical properties of Li, Na and K (sulfur). **g** Mechanism diagram of the functional separator interface for suppressing the shuttle effect

With the rapid development of society and ever-increasing energy consumption, the demand for renewable energy has reached a high level. The rechargeable battery systems (lithium/sodium/potassium ion batteries or supercapacitors) play an important role in electrochemical energy conversion and storage devices. Lithium‐ion batteries (LIBs) have been commercialized due to their high energy density, long cycle life, low self‐discharge, and minimal environmental pollution. However, commercial alkali (Li/Na/K) ion batteries (AIBs) rely on the reversible insertion-extraction of ions between cathode and anode, which limits their practical cell-level energy densities to about 200–500 Wh kg^−1^, insufficient to meet the increasing energy demands of modern applications [1–3]. Rechargeable M-S batteries, such as lithium-sulfur (Li–S) batteries, sodium-sulfur (Na–S) batteries, and potassium-sulfur (K–S) batteries, use cost‑effective, earth‑abundant sulfur cathodes, in contrast to conventional AIB cathodes built from scarce metals like cobalt, manganese or nickel (Fig. 1). On an active-material basis, M-S batteries can achieve much higher theoretical energy densities. For example, Li–S batteries possess a high specific capacity of 1675 mAh g^−1^ and a theoretical energy density of approximately 2600 Wh kg^−1^ based on sulfur. The practical cell-level performance of M-S batteries depends strongly on several critical factors, including the electrolyte-to-sulfur ratio (E/S), the negative-to-positive capacity ratio (N/P), and the areal sulfur loading (Fig. 2d, f). However, in Li–S batteries, the cost advantage of the sulfur cathode is offset by the combination with lithium, an expensive and geologically scarce anode material. (Industrial-grade lithium metal is about 100 $ kg^−1^, with a low abundance of approximately 0.0065% in the Earth's crust, and its concentration in seawater is about 0.17 mg L^−1^) In contrast, Na–S and K–S batteries leverage sodium and potassium resources, which are both inexpensive and abundant globally. (Industrial-grade sodium metal is 2–3 $ kg^−1^, with an abundance of approximately 2.36% in the Earth's crust and an extremely high content in seawater, about 10,620 mg L^−1^. Industrial-grade potassium metal is 3–5 $ kg^−1^, with a content of approximately 2.09% in the Earth's crust and about 380 mg L^−1^ in seawater.) [4–6]. It should be noted that the cost values in Table 1 refer to the prices of commonly used carbonate precursors (Li_2_CO_3_, Na_2_CO_3_, K_2_CO_3_), which are widely adopted as industry benchmarks for estimating raw material costs in battery manufacturing, rather than the direct prices of metallic lithium, sodium, or potassium [2]. Besides, we also summarize the corresponding crustal abundances, thereby providing a clear comparison of resource availability and raw material costs among the three alkali metal systems. Therefore, Na–S and K–S batteries emerge as desirable alternatives to conventional ion batteries, offering a more cost-effective edge and superior elemental abundance and material accessibility.

**Table 1:** Physical properties of Li, Na and K metal anodes [41, 42]

	Li	Na	K
Content in the Earth's crust (*wt*%)	0.0065	2.36	2.09
The price of metal carbonates ($ ton^−1^)	≈7400	≈141	≈360
Ionic radius (Å)	0.76	1.02	1.38
Stokes radius in PC (Å)	4.8	4.6	3.6
Desolventization energy in PC (kJ mol^−1^)	215.8	158.2	119.2
Conductivity of PC (S cm^2^ mol^−1^)	8.3	9.1	15.2
Potential *vs* SHE (V)	-3.04	− 2.71	− 2.93
Theoretical capacity of anode (mAh g^−1^)	3860	1165	685
Theoretical volume capacity of anode (mAh cm^−3^)	2062	1128	591
Sulfur-based theoretical energy density (Wh kg^−1^)	2567	1274	1023
Melting Point (°C)	180.5	97.8	63.5
Boiling Point (°C)	1342	883	759
Autoignition Temperature (°C)	179	115	54

However, their energy densities are constrained by multiple factors, especially the notorious shuttle effect, which is even more severe in Na–S and K–S systems due to the large ionic radii and weak solvation of Na^+^/K^+^. Moreover, sluggish redox kinetics and poor electrolyte/electrode compatibility further amplify this effect, resulting in rapid polysulfide migration, accelerated capacity fade and severe interface degradation. Cathode-side modified strategies have been widely investigated and proven effective at suppressing the shuttle effect [7–10]. Early approaches employ lightweight carbon hosts but suffer from limited sulfur loading. Subsequently, the design of nanostructured carbons with tailored porosity has achieved high sulfur contents above 70 *wt* % while maintaining high areal capacities. Introducing polar or catalytic mediators into the cathode matrix enables combined physical–chemical adsorption and accelerates polysulfide conversion, thereby improving reaction kinetics. However, ensuring homogeneous dispersion of these mediators without compromising electronic conductivity remains challenging, and balancing high sulfur loading with long-term mechanical integrity under repeated volume changes is essential to truly suppress the shuttle effect at scale.

In contrast, separator and interlayer-based interface engineering creates an active “second barrier” that more directly inhibits polysulfide shuttling and parasitic side reactions. By integrating a functional interface that encompasses ion selectivity, adsorption binding and catalytic conversion, the simultaneous physical capture and chemical binding of migrating polysulfides can be achieved, as well as a more uniform Li^+^/Na^+^/K^+^ flux across the battery. These low-cost, easily processed modifications significantly enhance cycling stability and coulombic efficiency at high sulfur loadings, and they reinforce the separator’s mechanical strength, effectively inhibit dendrite penetration and improve battery performance at low temperatures. Moreover, targeted control of the interface of separator and interlayer porosity and surface chemistry allows precise tuning of ion transport pathways and local electrochemical environments. These factors are critical for fast kinetics and long-term durability. Overall, the separator/interlayer interface engineering reduces polysulfide shuttling and battery impedance without causing significant mass gain or cost loss, laying the foundation for more powerful shuttle suppression. Building on these advances, solid‑state electrolytes (SSEs) show great promise as leak‑free barriers with intrinsically high mechanical modulus and negligible polysulfide solubility, offering a robust solution to shuttle‑induced capacity fade and interface degradation.

Although considerable efforts have been devoted to M-S batteries, a comprehensive review focusing on the inherent differences and connections in interface engineering for suppressing shuttle effects in alkali M (Li, Na, K)-S batteries is still scarce. While most previous reviews have primarily focused on individual battery systems, this work provides a comprehensive cross-system perspective by systematically comparing Li–S, Na–S, and K–S batteries. Through analyzing their distinct interfacial engineering, shuttle behaviors, and mitigation strategies, we aim to explore universal design principles from the common points of the three systems, while highlighting system-dependent optimization pathways, thereby complementing existing single-system studies. Herein, we will point out that the challenges faced by Li-, Na- and K–S batteries all stem from the inherent differences in the properties of these three metals. Based on the in-depth study of the Li–S system, we will further analyze and compare the Na–S and K–S systems to highlight the commonalities and individuality of each system. Among them, interface engineering is regarded as the core means to suppress the shuttle effect of polysulfides. The first part will discuss the characteristics of the sulfur cathodes, and then the similarities and differences in the reaction mechanisms among the three systems will also be compared. Subsequently, we will summarize the application of internal interface engineering in shuttle suppression. Starting from the separate-electrode interface, the intermediate layer (cathode and anode sides), and the SSEs, respectively, it systematically presents the design strategies and practical application effects of different interface strategies. Finally, we will look forward to the development direction of the next-generation alkali M-S batteries, with a focus on the functional interface design and its potential applications in the future energy storage and energy conversion fields.

## Sulfur Cathode and the Reaction Mechanism of Li/Na/K–S Batteries

Sulfur is renowned for its high natural abundance, low cost, and environmentally friendly, non-toxic properties. As the cathode material for alkali M-S batteries, sulfur boasts a theoretical capacity of 1672 mAh g^−1^, and its energy storage process is based on multi-electron redox reactions, which is distinct from the traditional ion intercalation/deintercalation reactions, allowing for higher capacity and energy output. Li–S batteries are approaching large-scale commercialization. However, reliance on costly and unevenly distributed lithium metal anodes has driven up costs. Given the characteristics of sodium and potassium mentioned above, Na–S and K–S batteries are expected to offer greater cost advantages.

### Li/Na–S Battery

The Na–S battery is capable of delivering an energy density of 1274 Wh kg^−1^. The earliest designs utilized molten sulfur for the cathode and molten sodium for the anode, with a β-alumina SSE interlayer (β-Al_2_O_3_) serving as both the electrolyte and separator. Since both the cathode and anode need to be in a molten state, the operation temperature of these batteries must be maintained above 300 °C, which inevitably increases operational costs and reduces safety [11, 12]. Consequently, researchers have turned their attention to room-temperature Na–S (RT Na–S) batteries [13]. While Na–S batteries share chemical similarities with Li–S batteries, they are not identical.

The working mechanism of Na (Li)-S batteries is essentially the redox reaction of sulfur elemental by obtaining or losing electrons in the electrochemical process. During the discharge process, the Na (Li) anode loses an electron and is oxidized to Na^+^ (Li^+^). The generated Na^+^ (Li^+^) would migrate from the anode to the sulfur cathode through the electrolyte. Then, it will combine with the sulfur and electrons transferred from the external circuit and form polysulfide intermediates as well as sodium/lithium sulfide (Na_2_S/Li_2_S) at the full-discharged state. In the consequent charging process, the Na_2_S (Li_2_S) was reversibly oxidized to elemental sulfur. The reversible oxidation from Na_2_S (Li_2_S) to sulfur features an extended charging plateau, with an initial voltage drop during the charging phase, indicating the overpotential required to drive the conversion of Na_2_S (Li_2_S) into polysulfide species. Concurrently, the metallic anode undergoes a reversible stripping/plating process during the repeated discharge/charge cycles. The corresponding reaction equation is as follows (Eqs. 1–3) (Metal, M = Li or Na):

1 $${\mathrm{Cathode}}{:}{\mathrm{S}}_{n} + 2{\mathrm{nM}}^{ + } + 2{\mathrm{ne}}^{ - } \leftarrow \to {\mathrm{nM}}_{2} {\mathrm{S}}$$

2 $${\mathrm{Anode}}{:}{\mathrm{M}} \leftarrow \to {\mathrm{M}}^{ + } + {\mathrm{e}}^{ - }$$

3 $${\mathrm{Overall}}\;{\mathrm{reaction}}{:}{\mathrm{S}}_{{\mathrm{n}}} + 2{\mathrm{nM}} \leftarrow \to {\mathrm{nM}}_{{2}} {\mathrm{S}}\left( {{1} \le {\mathrm{n}} \le {8}} \right)$$

#### Ether-Based Electrolytes

Contrary to the simplified equations previously mentioned, the actual electrochemical processes of Li/Na–S batteries involve a series of complex reactions, which can differ significantly across various electrolyte systems. In ether-based electrolytes, RT Na–S and Li–S batteries both undergo a transformation process from solid to liquid and back to solid [14]. In Li–S batteries, a typical constant current charge–discharge curve exhibits two discharge plateaus (Fig. 2a). The higher 2.3–2.4 V plateau corresponds to the opening of the cyclic octa atom sulfur ring (ring-S_8_), followed by the transformation of lithium polysulfide (LiPS) from a high-chain form to a low-chain (S_8_ → Li_2_S_8_ → Li_2_S_6_ → Li_2_S_4_), the contribution of theoretical specific capacity is 418 mAh g^−1^. The lower voltage plateau at about 2.0–2.1 V is associated with the reaction from the short-chain liquid Li_2_S_4_ to the solid Li_2_S_2_ and eventually to the equilibrium solid Li_2_S, thus constituting a residual capacity of 1255 mAh g^−1^. The detailed reaction equations are as follows (Eqs. 4–8):

4 $${\text{Stage I}}{:}{\mathrm{S}}_{{8}} + {\mathrm{2Li}}^{ + } + {\mathrm{2e}}^{ - } \to {\mathrm{Li}}_{{2}} {\mathrm{S}}_{{8}} \left( {{\mathrm{Platform}}:{2}.{3} - {2}.{4}\;{\mathrm{V}}} \right)$$

5 $${\mathrm{3Li}}_{{2}} {\mathrm{S}}_{{8}} + {\text{2 Li}}^{ + } + {\text{2 e}}^{ - } \to {4}\;{\mathrm{Li}}_{{2}} {\mathrm{S}}_{{6}}$$

6 $${\text{Stage II}}{:}{\mathrm{2Li}}_{{2}} {\mathrm{S}}_{{6}} + {\mathrm{2Li}}^{ + } + {\mathrm{2e}}^{ - } \to {\mathrm{3Li}}_{{2}} {\mathrm{S}}_{{4}}$$

7 $${\mathrm{Stage}}\;{\mathrm{III}}{:}{\mathrm{Li}}_{{2}} {\mathrm{S}}_{{4}} + {\mathrm{2Li}}^{ + } + {\mathrm{2e}}^{ - } \to {\mathrm{2Li}}_{{2}} {\mathrm{S}}_{{2}} \left( {{\mathrm{Platform}}{:}{2}.0 - {2}.{\mathrm{1V}}} \right)$$

8 $${\mathrm{Li}}_{2} {\mathrm{S}}_{2} + 2{\mathrm{Li}}^{ + } + 2{\mathrm{e}}^{ - } \to 2{\mathrm{Li}}_{2} {\mathrm{S}}$$

It is worth noting that the discharge process of Li/Na–S batteries is intrinsically multistep and involves several intermediate polysulfide transformations, each associated with a potential range rather than a fixed value. This is because the exact onset potential varies with electrolyte composition, catalyst type, and current density. Importantly, recent in-situ Raman and potentiostatic studies have revealed that even the so-called “first plateau” is not a single-step reaction, but rather a cascade of dissolution, fragmentation, and disproportionation events, and their potential windows overlap and shift dynamically during cycling [15, 16]. The above-mentioned mechanism and uncertainty also exist in Na–S and K–S systems. The larger ionic radii and weaker solvation often make the conversion step more difficult and lead to more polarization. Therefore, the strategy of catalyzing or interfacial regulation of Li^+^ (Na^+^/K^+^) activity often pushes the apparent step potential towards a more ideal range and enhances the platform's reversibility.

Furthermore, reported potentials are often derived under specific conditions and reflect a combination of thermodynamic and kinetic factors, including local cation activity, solvation structure, and interfacial overpotentials. For instance, Li^+^ is heavily solvated and weakly bound to polysulfides, while Na^+^ and K^+^ exhibit stronger electrostatic association with S_x_^2−^ chains, resulting in lower apparent potentials and larger hysteresis in Na–S and K–S batteries. These deviations imply that, when catalyzing the transformation process, catalytic activity should not be judged solely by absolute step potentials, but rather by its ability to lower overpotentials, accelerate Li_2_S_2_/Li_2_S conversion, and stabilize multistep transformations across the full discharge process [17].

Theoretically, RT Na–S batteries also convert S_8_ into Na_2_S through a multi-step reaction during the discharge process in ether-based electrolytes (Fig. 2b), and the specific discharge process is as follows (Eqs. 9–14):

9 $${\mathrm{Stage}}\;{\mathrm{I}}{:}{\mathrm{S}}_{8} + 2{\mathrm{Na}}^{ + } + 2{\mathrm{e}}^{ - } \to {\mathrm{Na}}_{2} {\mathrm{S}}_{8} \left( {{\mathrm{platform}}{:}\sim 2.2\;{\mathrm{V}}} \right)$$

$${\text{Stage II}}{:}{\mathrm{Na}}_{{2}} {\mathrm{S}}_{{8}} + {\mathrm{2Na}}^{ + } + {\mathrm{2e}}^{ - } \to {\mathrm{2Na}}_{{2}} {\mathrm{S}}_{{4}}$$ 1(10)

11 $${\mathrm{Stage}}\;{\mathrm{III}}{:}{\mathrm{3Na}}_{{2}} {\mathrm{S}}_{{4}} + {\mathrm{2Na}}^{ + } + {\mathrm{2e}}^{ - } \to {\mathrm{4Na}}_{{2}} {\mathrm{S}}_{{3}} \left( {{\mathrm{platform}}{:}\sim {1}.{\text{65 V}}} \right)$$

12 $${\mathrm{Na}}_{{2}} {\mathrm{S}}_{{4}} + {\mathrm{2Na}}^{ + } + {\mathrm{2e}}^{ - } \to {\mathrm{2Na}}_{{2}} {\mathrm{S}}_{{2}}$$

13 $${\mathrm{Na}}_{{2}} {\mathrm{S}}_{{4}} + {\mathrm{6Na}}^{ + } + {\mathrm{6e}}^{ - } \to {\mathrm{4Na}}_{{2}} {\mathrm{S}}$$

14 $${\mathrm{Stage}}\;{\mathrm{IV}}{:}{\mathrm{Na}}_{{2}} {\mathrm{S}}_{{2}} + {\mathrm{2Na}}^{ + } + {\mathrm{2e}}^{ - } \to {\mathrm{2Na}}_{{2}} {\mathrm{S}}$$

The larger radius of Na^+^ compared to Li^+^ results in lower reactivity with sodium polysulfides (NaPS), reducing their degree of dissociation and solubility in the electrolyte, and minimizing undesirable side reactions [18]. Based on the above explanation, K^+^ has the largest radius among the three ions, which results in the lowest solvation energy. At the same time, the nucleophilic reaction between potassium polysulfide (KPS) and solvents is also the weakest. These characteristics enable K–S batteries to demonstrate potential for stable operation in electrolytes, similar to Na–S batteries [19]. Moreover, K–S batteries may exhibit more stable conversion reaction characteristics.

#### Carbonate-Based Electrolytes

In carbonate-based electrolyte systems, free long-chain polysulfide anions in Li–S batteries can undergo vigorous nucleophilic addition or substitution reactions with the electrolyte, leading to the formation of thioethers and other sulfur-containing functional groups. This reaction may exhibit ultra-high discharge capacity in the initial cycles, even exceeding the theoretical specific capacity of sulfur [20]. However, the uncontrolled dissolution of LiPS leads to rapid capacity decay and reduced coulombic efficiency. Moreover, the poor compatibility of carbonate-based electrolytes with Li anodes may accelerate dendrite growth, increasing safety risks. As a result, carbonate-based electrolytes are less commonly used in Li–S batteries [21].

In contrast, sodium ion has more moderate chemical properties. Carbonate-based electrolytes are compatible with Na–S battery electrodes, providing a wide electrochemical window and high ionic conductivity [22–24]. Due to the availability, lower price, better thermal stability and industrialization basis of the carbonate electrolyte raw materials, Na–S battery shows higher application potential in large-scale, low-cost and high-safety energy storage scenarios [25, 26]. During this process, the sulfur directly converts into solid Na_2_S_2_ or Na_2_S, and the sulfur exists in the form of small molecules or interacts strongly with the matrix material, preventing the formation of soluble NaPS and thus suppressing the shuttle effect [20, 27]. Researchers have confirmed that Na_2_S is the sole discharge product under various discharge states by analyzing the first derivative curves of in situ ultraviolet–visible spectroscopy [28]. Since the rate of solid-to-solid transformation reactions is relatively slow, it can be accelerated by designing microporous structures or defect engineering through atomic doping to enhance internal ionic and electronic transport. Similarly to the addition of LiNO_3_ in the ether-based electrolyte of Li–S batteries, the addition of fluoroethylene carbonate (FEC) in carbonate-based electrolytes also helps to form a protective SEI layer, preventing excessive side reactions [29]. Overall, the low solubility of NaPS and the stable anode and cathode interfaces endow Na–S batteries with more stable cycling performance in carbonate-based electrolytes.

The intrinsically low solubility of NaPS, combined with the stable electrode–electrolyte interfaces, endows Na–S batteries with enhanced cycling stability in carbonate-based electrolytes. Importantly, the reaction mechanisms and interfacial behaviors of S/C composite and SPAN cathodes differ substantially, necessitating distinct SEI/CEI optimization strategies. For S/C composites, the conversion predominantly follows a solid–solid pathway owing to the extremely low NaPS solubility, which effectively mitigates shuttle effects but also slows redox kinetics. Recent studies demonstrate that introducing FEC facilitates the in situ formation of uniform NaF-rich CEI and SEI layers, which collectively enhance interfacial stability, improve redox reversibility, and extend cycling durability [30]. Functional additives and defect-engineered S/C hosts have been shown to accelerate solid–solid conversion and achieve superior rate performance. In contrast, SPAN cathodes, where sulfur is covalently anchored within the polymer backbone, inherently suppress the generation of soluble NaPS, rendering battery performance more dependent on the stability of the SEI at the Na anode. Recent advances show that engineering inorganic-rich SEI (e.g., NaF or Na_3_PO_4_) via FEC-containing electrolytes or weakly solvating electrolytes (WSEs) effectively suppresses dendrite formation, stabilizes Na deposition, and significantly improves cycling lifespan [31, 32]. These findings highlight that interfacial engineering in carbonate-based Na–S batteries should adopt cathode-specific strategies: S/C composites benefit from a coordinated CEI-SEI approach to facilitate solid–solid conversion, whereas SPAN cathodes demand SEI-centric stabilization to achieve long-term electrochemical stability.

### K–S Battery

Potassium‑based battery systems benefit from the weaker Lewis acidity of K^+^ in ether electrolytes, its relatively small Stokes radius in solution and high ionic mobility, all of which promote fast ion transport. It should be noted that the “stability” of potassium polysulfides mentioned earlier mainly refers to their low nucleophilicity toward electrolyte solvents, which effectively reduces parasitic interfacial reactions and enhances interface compatibility. By contrast, the thermodynamic stability of solid K_2_S_x_ phases represents another aspect: these crystalline intermediates are less prone to decomposition but introduce more complex multi-step conversion pathways during discharge. However, a K–S battery operates at a lower discharge voltage (~ 1.88 V) and suffers from a higher atomic mass of potassium (39 g mol^−1^), which together enables its theoretical specific energy at around 1023 Wh kg^−1^. Despite this, it is still about twice as high as the energy density of LIBs. The charge/discharge processes of the K–S battery also involve complex electrochemical mechanisms. Depending on the electrolyte composition, electrode materials, and structural design, the final discharge products may be K_2_S_3_, K_2_S_2_, or K_2_S. Generally, the transformation process in most K–S batteries can be summarized as the stepwise reduction of sulfur to long-chain polysulfides, then to short-chain polysulfides, and ultimately to K_2_S_n_ (1 ≤ n ≤ 3). Unlike the unstable polysulfides in the Li/Na–S battery, the KPS have a series of stable phases at room temperature (K_2_S_n_, n = 1,2,3,4,5,6), which makes it more convenient for researchers to use various possible characterization techniques in future studies to explore its mechanism further [33, 34].

Theoretically, due to the large ionic radius and slow reaction kinetics, each phase transition from K_2_S_n+1_ to K_2_S_n_ (1 ≤ n ≤ 7) generates a corresponding platform, which is reflected on the charge–discharge curve as a gently inclined profile with a certain gradient (Fig. 2c). Similar to the Li/Na–S battery, the working reactions of the K–S battery can be described as Eq. 15:

15 $${\mathrm{xS}} + 2{\mathrm{K}} \leftarrow \to {\mathrm{K}}_{2} {\mathrm{S}}_{{\mathrm{x}}} \left( {1 \le {\mathrm{x}} \le 3} \right)$$

In early studies, sulfur was encapsulated within CMK-3 mesoporous carbon as the active material, and XRD measurements indicated that long-chain polysulfides ultimately transformed into K_2_S_3_ [35]. However, the theoretical capacity of K_2_S_3_-K_2_S accounts for 67% of the total S capacity (1675 mAh g^−1^), so it is crucial to completely reduce K_2_S_n_ to K_2_S through a reasonable strategy for battery capacity improvement. It has been concluded that the reduction of K_2_S_3_ to K_2_S is not directly formed, but through proportionation and comproportionation reactions between polysulfides [36]. Xiong et al. pointed out that K_2_S_2_ tends to form K_2_S_3_ and K_2_S because the formation energy of K_2_S_2_ is higher than that of K_2_S_3_ and K_2_S (in terms of stability, K_2_S_3_ ≥ K_2_S ≥ K_2_S_2_) based on theoretical density functional theory (DFT) calculations [37]. Sun et al. achieved full solid‑state S → K_2_S conversion in a SPAN electrode using a polyacrylic acid binder, demonstrating the effectiveness of covalent-S. Beyond this approach, atomic catalysis strategy (e.g., single‑atom Co, Ni, Fe, Mn, or Cu in N‑doped carbon) also drives the K_2_S_3_ → K_2_S step by tuning metal-sulfur bond strength, lowering overpotentials and accelerating kinetics [38, 39]. Taken together, these strategies balance atomic configuration and electronic structure, providing a clear blueprint for designing advanced sulfur cathode catalysts.

Although we have borrowed extensively from the mature mechanistic framework developed for Li–S batteries and achieved promising results, a comprehensive understanding of conversion pathways in Na–S and K–S batteries will demand further ingenuity. By combining in-situ techniques (e.g., operando spectroscopy platforms and in-situ TEM) with advanced synthesis and modeling, we can capture the true speciation and structural evolution of sulfur species throughout cycling. Conventional ex-situ analyses alone cannot resolve the transient intermediates formed during charge and discharge. For example, Hu et al. combined in‑situ XRD and ex‑situ XPS to reveal the reversible S_8_ ↔ K_2_S/K_2_S_2_ conversion in a K–S battery [40]. Guided by DFT screening, they designed an N‑doped carbon-WSA-W_2_C host that both lowers the dissociation barrier of solid KPS and accelerates polysulfide conversion. This mechanism‑driven “design‑experiment‑validate” approach not only deepens our understanding of sulfur conversion but also provides a clear blueprint for next‑generation M-S battery materials.

## Internal Interface Management System in Preventing Shuttle Effects

As described in preceding sections, Li/Na/K–S batteries universally grapple with the detrimental polysulfide shuttle effect. In Li–S batteries, extensive research has been conducted on functionalized separators and interlayers that incorporate polar materials or catalytic sites to immobilize and convert polysulfides effectively. These strategies have been adapted to Na–S batteries, yielding promising results. However, K–S batteries present unique challenges due to the larger ionic radius of K^+^ and its distinct electrochemical behavior, requiring a customized interface design. The physicochemical differences among Li, Na and K (ionic radius, solvation dynamics and redox potential) drive distinct polysulfide chemistries and shuttle behaviors (Table 1). Therefore, while interfacial engineering principles may be universally applicable, their specific implementations must be tailored to each system. The subsequent sections will delve into comparative analyses of separator/interlayer modifications and SSEs design across Li–S, Na–S, and K–S batteries, elucidating both shared strategies and system-specific adaptations.

### Separator Interface Modification

#### Design Principle for Functionalized Separator Engineering

Polyolefin separators, such as polypropylene (PP) and polyethylene (PE), are widely employed in Li–S batteries due to their low cost, electrochemical stability, suitable mechanical strength, and appropriate porosity. These separators allow sufficient electrolyte uptake and ion diffusion while physically separating the cathode and anode to prevent internal short circuits. However, conventional carbonate-based electrolytes often exhibit poor wettability with polyolefin membranes, and the larger ionic radii of Na^+^ and K^+^ demand wider ion transport channels to enable efficient migration [43]. Consequently, Na–S and K–S batteries commonly employ glass fiber (GF) separators, which offer higher porosity and better electrolyte wettability, particularly with carbonate solvents such as EC and PC that provide high sodium salt solubility [44, 45]. However, conventional GF separators suffer from large micron‑scale pores and excessive thickness, which increase electrolyte uptake, lower energy density, and heighten safety risks with flammable solvents. Besides, their high porosity also accelerates polysulfide migration, while the large pores allow dendrite penetration that degrades coulombic efficiency and can cause short‑circuits [46, 47].

To quantitatively assess separator ionic conductivity, the following relation is commonly used (Eq. 16):

16 $$\sigma =\frac{L}{R\cdot A}$$

where σ is the ionic conductivity (S cm^−1^), L is the membrane thickness (cm), R is the measured bulk resistance (Ω), and A is the electrode contact area (cm^2^). High porosity and good wettability contribute to a lower R, thus enhancing overall ionic conductivity.

Furthermore, to evaluate the separator's cation transport selectivity, particularly critical in suppressing polysulfide anions, the cation transference number t^+^, is determined via the Bruce-Vincent-Evans method with the cells assembled using a metal /metal symmetrical cell structure (Eq. 17):

17 $${t}_{+}=\frac{{I}_{ss} (\Delta V-{I}_{0}{R}_{0})}{{I}_{0}(\Delta V-{I}_{SS}{R}_{ss})}$$

where I_0_ and I_ss_ are the initial and steady-state currents measured by chronoamperometry under a small polarization potential (10 mV) and the test time was set as 10,000 s, R_0_ and R_ss_ are the interfacial resistances before and after polarization, and ΔV is the applied DC bias. Furthermore, the test temperature can be adjusted according to the actual situation. A higher t_+_ value indicates enhanced cation-selective transport, which is beneficial for mitigating polysulfide shuttling, as summarized in Table 2.

**Table 2:** Electrochemical performance summary of various ion-selective interfaces for M-S batteries

Separator type	Battery type		layer thickness (material loading)		Sulfur loading (mg cm^−2^)	E/S ratio	N/P ratio	t^+^	Practical capacity (mAh g^−1^) (Rate)	Cycling performance (mAh g^−1^) (Cycles/Rate)	Capacity decay (%)	References
MX@NF/PP	Li–S		0.2 mg cm^−2^		2.0	NA	NA	NA	920 (1 C)	645 (1000/1 C)	0.03	[90]
SPEEK-100	Li–S		0.4 mg cm^−2^		6.0	NA	~ 14.36	NA	1000 (0.2 C)	550 (300/0.2 C)	0.18	[61]
LNPA	Li–S		15 µm		~ 1.0	31.6	~ 61.6	0.72	1272 (0.1 C)	788 (1000/1 C)	0.022	[62]
MOF/Nafion	Li–S		34 μm		1.0	~ 50	NA	0.66	594 (0.5 C)	534.5 (300/0.5 C)	0.03	[59]
CNT/Nafion/PEDOT:TCB	Li–S		750 nm (0.182 mg cm^−2^)		3.4	20	NA	NA	1113 (0.2 C)	797 (100/0.2 C)	0.055	[63]
CTP-PAN-LLZTO	Li–S		CTP (~ 100 nm)		3.5	~ 7–15	NA	NA	1287.9 (0.5 C)	905.5 (500/0.5 C)	0.059	[64]
COF-TpPa@2400	Li–S		50 μm		1.0–1.5	~ 25–40	NA	0.62	1636.4 (0.1 C)	1145.5 (100/0.1 C)	0.3	[65]
Na-Nafion	Na–S		0.5 μm		~ 2.0	NA	NA	NA	465 (0.1 C)	350 (20/0.1 C)	0.012	[66]
CNF/AC	Na–S		~ 50 μm		2–3	~ 60–70	NA	NA	1200 (0.05 C)	550 (100/0.2 C)	0.35	[67]
Na-Nafion/AC-CNF	Na–S		30 μm (0.2 mg cm^−2^)		~ 2.2	~ 25–30	NA	NA	790 (0.2 C)	550 (100/0.2 C)	0.3	[58]
Al_2_O_3_-Nafion	Na–S		~ 8.5 μm		NA	NA	NA	NA	350 (0.1 C)	250 (100/0.1 C)	0.29	[68]
HB/CNT@COF	Na–S		1.5 µm		0.5 ~ 1.0	10	~ 5	0.78	1133 (0.1 C)	733.4 (400/4 C)	0.021	[69]
PBI	Na–S		8.0 μm		1.0 ~ 1.5	65 ~ 100	~ 10 ~ 30	NA	837 (0.1 C)	650 (50/0.2 C)	0.14	[70]
Azo-TbTh COF	Na–S		70 nm		1.0	100	~ 33.7	0.89	1295 (0.2 C)	524 (1000/1 C)	0.036	[71]

In Li/Na/K–S batteries, designing functional interfaces with advanced materials has proven effective in alleviating polysulfide dissolution, suppressing side reactions at the metal anode, and improving rate performance and cycle life. Functionalized separator surfaces, whether applied as direct coatings on commercial membranes or used as independent freestanding interlayers, typically incorporate components that adsorb, immobilize or catalytically convert polysulfides, reducing their solubility and accelerating redox kinetics to extend cycle life [48].

Functionalized separators must meet three basic criteria: (1) electrical insulation to prevent short circuits; (2) fast ionic transport to maintain electrochemical kinetics; and (3) electrochemical stability across the operational voltage range [49, 50]. It is worth noting that in the pursuit of transitioning alkali M-S batteries from laboratory to practical energy storage devices, achieving stable electrochemical performance under high sulfur loading and lean electrolyte conditions has emerged as a vital, yet critically challenging research focus. High sulfur areal loading is a prerequisite for attaining competitive energy densities that meet commercial application requirements. Concurrently, operating with a low E/S ratio is a crucial strategy for minimizing overall battery cost and maximizing the practical energy density at the cell level. However, high loading exacerbates the total mass of polysulfides susceptible to shuttling and intensifies mechanical strain within the electrode structure, while lean electrolyte severely deteriorates ion-transport kinetics and elevates the local concentration of polysulfides, making the shuttle effect more acute. Consequently, evaluating the efficacy of any interfacial engineering strategy (eg, cathode host, separator modifications, or binder systems) under the dual constraints of high sulfur loading and lean electrolyte has become one of the most significant benchmarks for assessing its practical viability [51–53].

High sulfur loading significantly increases the total amount of soluble polysulfide intermediates, which requires the separators to have excellent polysulfide blocking and absorbing capacity. For instance, when designing functional modification layers, high-density chemical adsorption sites (such as polarized surfaces, defect-engineered matrices, or atomic-level catalysts) should be laid out to effectively fix a large amount of polysulfides. Meanwhile, the situation of poor electrolytes poses a challenge to the resistance of ion transport. Functionalized separators should exhibit precise ion-selectivity that efficiently blocks polysulfides while maintaining low-energy-barrier pathways for rapid alkali metal ion transport. This balance is essential to avoid significant polarization that would otherwise negate the benefits of high-loading electrodes. Recent pioneering research underscores that the critical solution to these strict conditions lies in the rational design and preservation of a robust and functional active material microenvironment (ME@AM) [54]. In this context, Yan et al. demonstrated an alveoli-inspired carbon cathode with interconnected hierarchical pores and asymmetrically coordinated V-S_1_N_3_ single-atom sites, which synergistically enhance polysulfide adsorption and catalytic conversion through tilted adsorption configurations and optimized electronic structures. This design achieves ultralow conversion barriers (~ 0.21 eV) and long-term cycling stability, providing a representative framework for constructing multifunctional ME@AM architectures [55]. An ideal ME@AM should integrally provide mechanical resilience to withstand volume changes, ensure efficient and continuous pathways for ion and electron transport even in dense electrodes, and offer strong capabilities for polysulfide confinement and catalytic conversion, thereby rescuing battery performance under these stringent conditions. Beyond these, properties such as porosity, thickness, electrolyte wettability, and surface energy significantly influence overall battery behavior. As a result, research has increasingly focused on tailoring the separator interface with features such as ionic selectivity, strong polysulfide affinity, and catalytic activity. Integrating these parameters into a unified design strategy is crucial for advancing high-performance separators in alkali M-S battery systems (Fig. 2g).

#### Approaches to High-efficiency Separator Interface Management System

Recent advances in separator engineering have led to a wide range of modification strategies, including polymer coatings, 2D layered membranes (MXenes, graphene, etc.), MOF/COF frameworks, Janus separators, and composite nanostructures, etc. These diverse materials can be broadly classified based on their dominant interfacial functionality: selective ion transport, strong polysulfide adsorption, and catalytic acceleration of conversion reactions. Ion-selective separators utilize sub-nanometer pores or charged channels to selectively allow alkali-ion transport while physically blocking polysulfide species. Their key advantage is high efficiency at minimal design complexity, but the precise pore size control required can lead to compromised ionic conductivity at high sulfur loadings. Strong adsorption interfaces immobilize soluble polysulfides via polar surface groups or MOF/COF coatings, effectively suppressing shuttling. However, excessive adsorption can slow reaction kinetics and lower sulfur utilization, especially under lean-electrolyte conditions. Catalytic functional interfaces employ doping-defect engineering, single-atom catalysts, or d-band-engineered transition-metal sites to accelerate polysulfide conversion and improve redox kinetics, which significantly enhances cycling stability and high-rate performance, but catalyst cost and synthetic complexity may limit scalability. Overall, hybrid designs integrating ion selectivity, chemical adsorption, and catalytic acceleration are emerging as promising pathways, offering synergistic control of shuttle suppression and redox dynamics for next-generation high-performance Li/Na/K–S batteries.

##### Customs Inspection Post: Ion-selective Interface

The ion-selective membrane can be analogized to a customs checkpoint, where it selectively permits certain ions to pass while restricting or blocking others. Specifically, it facilitates the passage of alkali metal cations while simultaneously acting as a barrier to polysulfides. This selective permeability prevents the diffusion of polysulfides, effectively confining them within the cathode region and thus minimizing active material loss. As a result, the separator plays a critical role in enhancing sulfur utilization and improving the overall performance and cycle stability of M-S batteries.

*Electrostatic Repulsion*

**Fig. 3:**
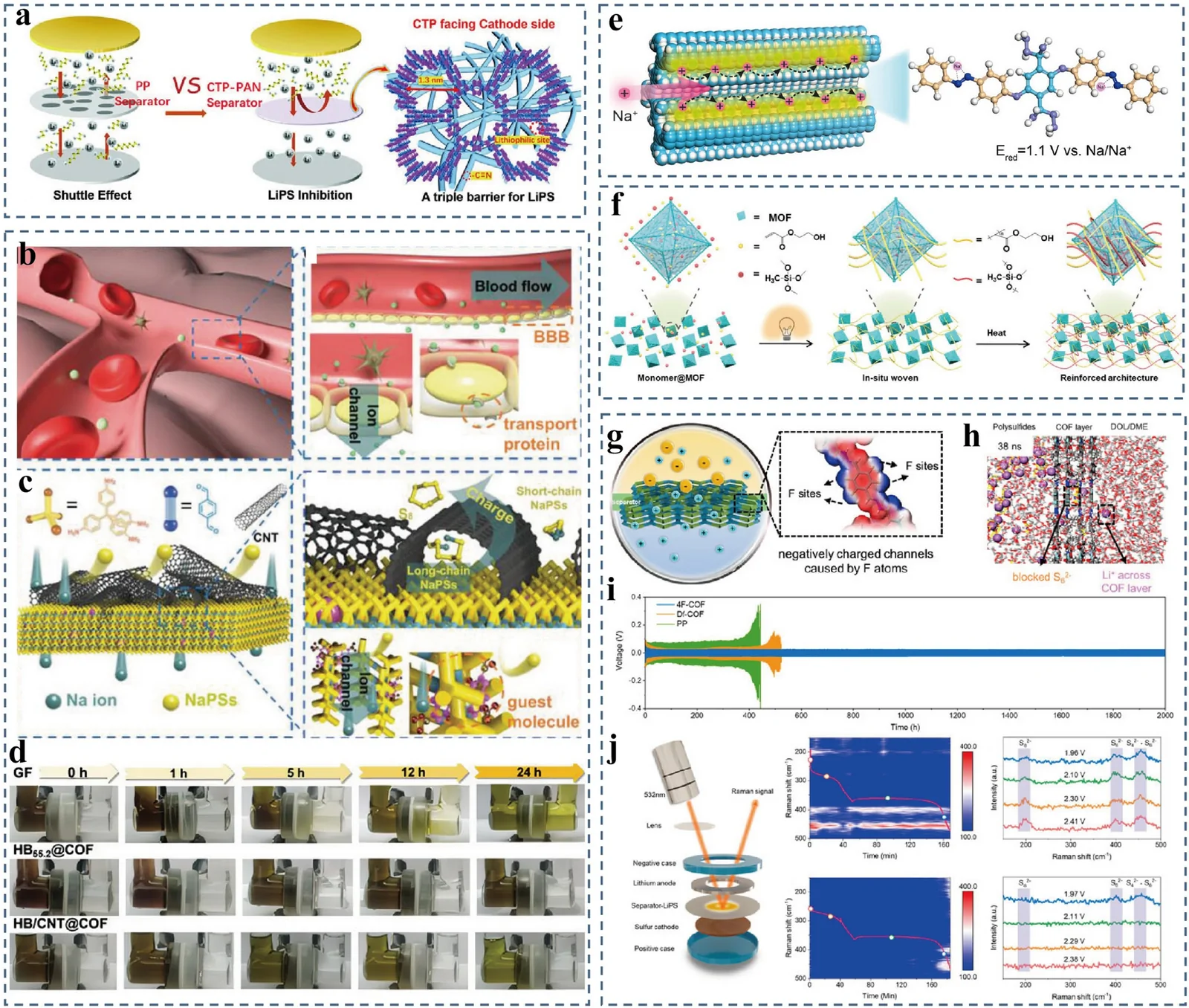
Schematic illustration of **a** the shuttle effect suppression with CTP-PAN separator; Reproduced with permission [64]. Copyright 2022, Wiley–VCH. **b** the flow of substances in cerebral vessels and the selective passage of ions through the BBB and the rejection of neurotoxins; **c** the selective Na^+^ transport and the use of HB/CNT@COF to repel polysulfides; **d** the permeation test of different membranes; Reproduced with permission [69]. Copyright 2023, Wiley–VCH.** e** the fast Na^+^ migration within the Azo-TbTh separator; Reproduced with permission [71]. Copyright 2022, American Chemical Society.** f** the two-step PHAP method; Reproduced with permission [75]. Copyright 2021, Wiley–VCH.** g** COF-based nanofluidic membrane **h** ion transport behavior through 4F-COF nanofluidic nanochannels. **i** Cycle stability of Li|Li symmetric cells based on different separators at 1 mA cm^−2^. **j** In situ time-resolved Raman spectra obtained during discharging processes with PP (top) and 4F-COF/PP (bottom) separators. Reproduced with permission [76]. Copyright 2023, American Chemical Society

One of the most typical ion-selective membranes is the Nafion membrane. This material can be specifically designed for various types of cations, and the surface with negative sulfonic acid groups can have a repulsion effect on anions. Nafion membranes, as a copolymer of tetrafluoroethylene and perfluorovinyl ether, have been widely used in energy storage devices such as fuel cells and flow batteries due to their excellent stability and high cationic conductivity [56, 57]. In Li/Na/K–S batteries, polysulfides dissolved in organic electrolytes will undergo ionization (M_2_Sn → 2 M^+^ + Sn^2−^, M = Li, Na, K, 4 ≤ n ≤ 8) or dissociation processes (S_n_^2−^ → 2 ${S}_{n/2}\cdot$^−^). Therefore, long-chain polysulfides are negatively charged [58]. In this way, Nafion contains perfluorinated backbones and sulfonic acid side chains, which offer strong electrostatic repulsion against negatively charged polysulfide species, thereby effectively mitigating their migration toward the metal anode [59, 60]. Nafion membranes have been effectively employed in Li–S battery research to modify separator interfaces and suppress polysulfide anion migration. Babu et al. conducted an early study combining Nafion with sulfonated PEEK, showing that a 1:1 ratio effectively blocked polysulfide crossover while maintaining ionic conductivity [61]. Building on this, He et al. developed a dual-layer separator with lithiated Nafion facing the cathode and porous Al_2_O_3_ on the anode side to simultaneously inhibit polysulfide shuttling and lithium dendrites [62]. Expanding Nafion’s functionality, Diao et al. integrated it with a Cu-metal–organic framework (Cu-MOF) ion-sieve membrane that regulated Li^+^ flux and restricted polysulfide diffusion through both steric hindrance and coulombic exclusion [59]. More recently, Son et al. fabricated a 750 nm ultrathin Nafion interlayer embedded in a CNT/PEDOT matrix using solution shearing. The resulting Li–S pouch cells delivered 1029 mAh g^−1^ at high sulfur loading (5.3 mg cm^−2^) and low E/S ratio (5 μL mg^−1^), showing excellent rate capability and cycling performance [63]. To further enhance energy density, binder-free, interfacially engineered Janus separators were prepared, which presented LiPS-trapping functionality and dendrite suppression (Fig. 3a) [64, 65].

In early Na–S battery studies, sodified Nafion (Na-Nafion) was drop-cast onto porous PP membranes to create cation-selective separators, resulting in a 75% capacity increase after 20 cycles [66]. Similarly, Manthiram et al. soaked Nafion membranes in a TEGDME electrolyte with NaClO_4_ and NaNO_3_ to obtain Na-Nafion, which was directly used in battery assembly without drying. This modification effectively suppressed NaPS shuttling and enhanced Na^+^ transport [67]. Building on this, they further coated Na-Nafion with an activated carbon nanofiber layer, serving as a secondary current collector and improving electrochemical performance. Efforts to improve sulfur utilization have also led to Al_2_O_3_-Nafion composite GF membranes via screen printing. These membranes exhibit polysulfide adsorption and deliver enhanced cycling stability, retaining about 250 mAh g^−1^ after 100 cycles [68]. In contrast, the use of Nafion membranes in K–S batteries remains largely unexplored. Considering the larger ionic radius of K^+^ and the distinct operating conditions of K–S systems, it is important to evaluate membrane compatibility. Future research could draw inspiration from modification strategies employed in Li/Na–S batteries, such as introducing nanoparticles, adjusting the film thickness or combining with other materials, to optimize their performance in K–S batteries.

In addition to Nafion membranes that utilize anionic groups to repel polysulfides, Chen et al. developed a hydroxynaphthol blue (HB)-modified single-walled carbon nanotube (SWCNT) composite membrane encapsulated with covalent organic framework (COF)-300 (HB/CNT@COF). The sulfonic acid groups in the HB dye provide effective ionic sieving capabilities [69]. Therefore, the permeation test proved that HB/CNT@COF membrane has superior blocking ability against polysulfides (Fig. 3d). Besides, the interpenetrating 3D network formed by the COF and conductive carbon nanotubes facilitates rapid ions/electrons transport, thereby reducing interfacial resistance caused by inert polysulfide layers. Drawing inspiration from the blood–brain barrier in medicine, which selectively regulates ion transport through negatively charged surfaces (Fig. 3b-c). Ren et al. introduced a polybenzimidazole-based ion-selective separator combined with a dual-function NaI-P_2_S_5_ electrolyte additive [70]. This design effectively stabilized the deposition of insoluble Na_2_S_2_ by controlling ion transport and precipitation reactions, achieving a capacity retention of 92.9% after 50 cycles in Na–S batteries. Wang et al. synthesized an azobenzene-functionalized COF thin film via interfacial polymerization and applied it as a modified GF separator in Na–S batteries [71]. The azobenzene side chains enabled sub-nanometer pore control and electrostatic repulsion, effectively suppressing polysulfide shuttling while facilitating Na^+^ transport through rapid ion migration channels (Fig. 3e). The ultrathin 70 nm film blocked polysulfide crossover without compromising ion transport kinetics, resulting in a high specific capacity of 1295 mAh g^−1^ at 0.2 C and excellent cycling stability (0.036% capacity decay per cycle over 1000 cycles at 1 C). Flexibility tests further confirmed its mechanical robustness. Although COF materials are relatively more expensive than traditional polymer coatings, ultra-thin COF layers have excellent Na^+^ selectivity, high ionic conductivity and extremely low capacity decay. These findings emphasize the trade-off between material costs and performance enhancements. This work underscores the potential of functionalized framework materials in separator design, offering promising strategies for advanced Na–S battery development.

*Customized Membrane Pore Size*

Traditional polyolefin separators suffer from uneven pore distribution, low polarity, and poor porosity, which limit their ability to block polysulfide diffusion. To address these issues, recent efforts have focused on designing selective membranes with tailored pore structures [72, 73]. For example, Zhao et al. developed an electrospun PI-PEO copolymer membrane that improved electrolyte wettability and ionic conductivity, while polar functional groups enhanced polysulfide affinity [74]. Functional porous materials such as MOF and COF have also shown great promise due to their ordered channels, charge selectivity, and chemical adsorption capabilities. Lan’s group introduced a MOF-based separator with stepped channels, enabling both polysulfide blocking and efficient Li^+^ transport (Fig. 3f), which led to a high capacity (1365 mAh g^−1^) and stable cycling over 700 cycles [75]. Similarly, a fluorinated covalent organic framework (4F-COF)-based separator was designed to create negatively charged nanochannels to achieve the same effect (Fig. 3g-i). In situ Raman spectroscopy further confirmed that the 4F-COF-modified separator effectively suppressed polysulfide migration, showing only weak polysulfide signals at the separator/lithium interface compared to the pristine PP separator. (Fig. 3j). Electrochemical measurements further revealed that the 4F-COF separator delivers a high Li^+^ transference number (0.73), which is significantly higher than that of pristine PP separators (0.33), ensuring efficient ion-selective transport and mitigating concentration polarization. This separator shows excellent electrochemical performance, including a cycling retention of 82.3% over 1000 cycles at 2 C and a rate performance of 568 mAh g^−1^ at 10 C, making it a promising solution for practical applications of Li–S batteries (Table 2) [76]. In Na–S systems, as we mentioned in the previous section, Wang et al. utilized azobenzene-functionalized COFs to achieve sub-nanometer pore control and uniform Na^+^ transport [71]. Composite ion-selective polymer membranes with interconnected pores further suppressed NaPS dissolution and enhanced Na^+^ conductivity (~ 10^–3^ S cm^−1^), offering a practical solution for RT Na–S batteries [77, 78]. Such membrane designs also help alleviate dendrite formation by promoting homogeneous ion flux, providing a promising direction for advanced M-S battery development. Although the application of COF has not been reported in K–S batteries yet, it has shown significant advantages in zinc-sulfur (Zn–S) battery systems [79]. An ionic COF (iCOF) membrane with negatively charged nanochannels effectively blocks S_8_/S_x_^2−^ while maintaining fast ion conduction (t^+^: 0.15, ionic conductivity: 18.22 mS cm^−1^). As a result, Zn–S batteries deliver 699.3 mAh g^−1^ at 1.0 A g^−1^, 175 mAh g^−1^ at 5.0 A g^−1^, and 92.4% capacity retention after 300 cycles. These results demonstrate that performance enhancement arises from the synergistic effect of shuttle inhibition and improved Zn^2+^ transport [30, 80]. In the future, the verification method combining DFT calculation and experiments will provide a methodological reference for the development of K–S battery separators. Recent studies emphasize the importance of nano/microstructural optimization in enhancing sulfur utilization. Li’s team reported that preferential Li_2_S precipitation on the cathode surface causes pore blockage and premature discharge termination [81]. Introducing the Thiele modulus provides a quantitative guideline, showing that optimizing porosity and pore connectivity effectively alleviates diffusion limitations and enables an energy density of 436 Wh kg^−1^ in a 3 Ah pouch cell. These findings underscore the significance of transport-aware structural design for improving practical sulfur kinetics.

To date, research on improving K–S battery performance through ion-selective separators remains scarce. Conventional separators often suffer from poor electrolyte wettability and uncontrolled pore size distribution, which induce inhomogeneous K^+^ flux and aggravate polysulfide shuttling, leading to rapid capacity decay. Therefore, developing advanced ion-selective separator architectures that simultaneously enable efficient K^+^ transport and effective polysulfide suppression is a promising strategy for achieving stable and long-term cycling in K–S batteries.

The efficacy of size-sieving separators critically depends on achieving an optimal pore size window determined by the effective dimensions of polysulfide species. Recent experimental and computational studies demonstrate that LiPS in typical ether-based electrolytes predominantly exist as solvated clusters rather than isolated chains. Molecular dynamics simulations, XRD, and in situ spectroscopic analyses have revealed dominant hydrodynamic diameters of approximately 0.6–0.9 nm, while longer-chain species (Li_2_S_6_/Li_2_S_8_) can occasionally form larger solvated aggregates up to ~ 1.2 nm [82–84]. For NaPS/KPS, direct experimental measurements remain limited. However, due to weaker solvation and stronger cation-polysulfide association, NaPS and KPS are inferred to form more compact ion-polysulfide aggregates or even gel-like phases, yielding an estimated effective size of ~ 0.7–1.5 nm under similar solvent conditions [85]. Based on these cluster size estimates, the optimal pore size for physical sieving should be designed slightly smaller than the polysulfides to block their diffusion while remaining larger than solvated alkali cations to minimize ion-transport impedance. Representative studies suggest an effective design window of approximately 0.45–0.60 nm for Li–S, 0.50–0.75 nm for Na–S, and 0.60–0.85 nm for K–S systems [86, 87]. In practice, these ultra-microporous apertures are commonly integrated as a “skin layer” atop a mesoporous scaffold (2–20 nm), enabling efficient polysulfide rejection while maintaining high ionic conductivity [82, 88, 89].

Although ion-selective interfacial strategies improve sulfur utilization and battery stability by mitigating the polysulfide shuttle effect, they still face significant limitations. By employing electrostatic repulsion to confine polysulfides within the cathode region, these strategies reduce anode corrosion. However, this confinement leads to elevated polysulfide concentrations near the cathode, increasing electrolyte viscosity and decreasing ionic conductivity. These effects are particularly detrimental under high sulfur loading, high current densities, and low-temperature conditions, resulting in significant performance degradation. Moreover, the accumulation of polysulfides at the cathode interface can form passivation layers, increasing interfacial resistance and further compromising cycling efficiency and stability. Consequently, while ion-selective membranes offer short-term benefits in suppressing polysulfide migration, their efficacy diminishes over prolonged cycling and under rigorous operational conditions. Size customization alone cannot balance complete polysulfide blocking with low-resistance ion transport, thus polar adsorption and catalytic conversion must be integrated for synergistic control. Future research should focus on developing multifunctional composite membranes with optimized interfacial designs and dynamic regulation capabilities. Enhancing mechanical strength, conductivity, and environmental adaptability will be crucial to overcoming challenges associated with membrane thickness and increased viscosity, thereby advancing the performance of alkali M-S batteries.

#### Molecular “Trap”: Strong Adsorption Function Interface

To overcome the intrinsic limitations of ion-selective membranes, a more proactive approach involves introducing chemically selective adsorption sites that act as molecular traps for long-chain polysulfides. By tailoring surface chemistry and binding strength, such functional interfaces immobilize soluble polysulfides at the cathode side, thereby reducing shuttle effects, enhancing sulfur reutilization, and improving cycling stability.

##### Carbon Materials

Carbon materials with high surface area, good conductivity and tunable pore structures were first employed by the Arumugam Manthiram group to modify Li–S battery separators, physically trapping soluble polysulfides and enhancing electrochemical performance [91, 92]. The shuttle-suppressing effect in Li–S batteries has been widely demonstrated, and carbon coatings have likewise shown efficacy in Na/K–S systems [58, 68, 93]. For example, a SWCNT layer on K–S battery separators markedly reduced KPS shuttling and improved sulfur utilization, resulting in a retained capacity of about 600 mAh g^−1^ after 50 cycles, which is significantly improved cycling stability compared with the unmodified battery [94]. Pristine carbon materials trap polysulfides through weak van der Waals interactions, offering excellent conductivity for redox reactions but insufficient suppression of the shuttle effect. In contrast, chemical adsorption by covalent bonding accelerates polysulfide conversion. To address this, modification strategies borrowed from Li–S systems have been adopted: heteroatom doping (N, O, S, P, etc.) to establish strong interfacial coupling [95–98]; polar metal oxides, sulfides, or nitrides to introduce active binding sites; and metal–organic chelates that leverage Lewis acid–base interactions for tight polysulfide confinement [99–101].

**Fig. 4:**
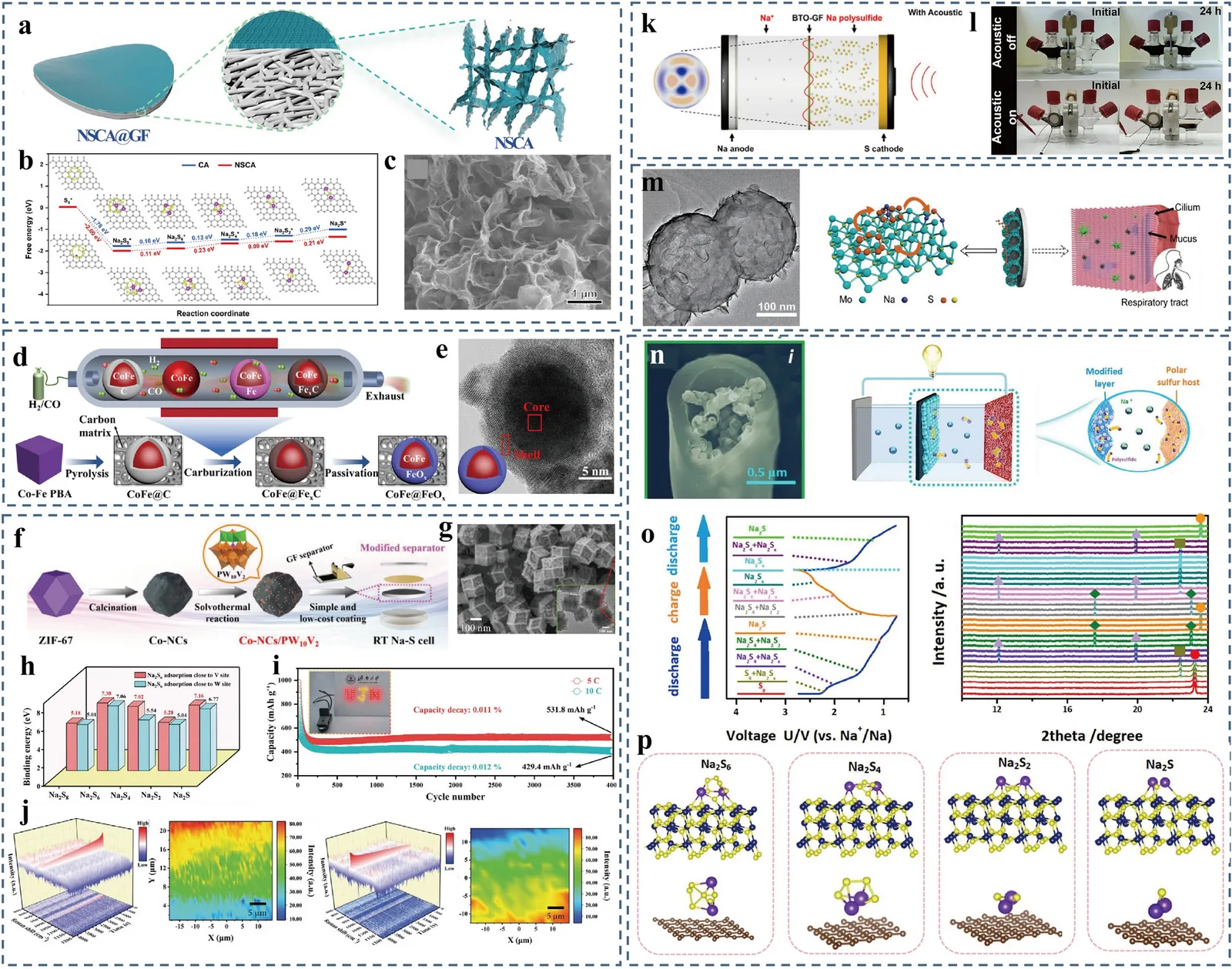
**a** Preparation process and morphological characterization of NSCA. **b** Gibbs free energy profiles. **c** SEM image. Reproduced under terms of the CC-BY license [106]. Copyright 2023, The Authors, published by Wiley.** d** Formation process of the CoFe@FeO_x_. **e** STEM image. Reproduced under terms of the CC-BY license [111]. Copyright 2023, The Authors, published by Springer Nature.** f** Synthesis process of the Co-NCs/PW_10_V_2_ modified separator. **g** SEM images (inset: TEM image). **h** Comparison of binding energies between various Na_2_S_n_ and PW_10_V_2_. **i** Long-cycling performance; **j** In situ electrochemical Raman characterization and mapping of the intensity of PW_10_V_2_ during charging (left) and discharging (right) process. Reproduced with permission [113]. Copyright 2024, Wiley–VCH. Schematic illustration of **k** BTO separator;** l** Visual observation of NaPS diffusion. Reproduced with permission [114]. Copyright 2024, American Chemical Society. **m** Absorption function and TEM images of the HCS/MoS_2_. Reproduced under terms of the CC-BY license [119]. Copyright 2019, The Authors, published by Wiley–VCH.** n** Hybrid fiber after etching (left) and the dual-polysulfide-defending system in RT-Na/S battery (right); **o** the initial charge/discharge curves (left) and corresponding XRD patterns (right); **p** DFT calculations. Reproduced with permission [120]. Copyright 2021, Elsevier

Building on these principles, Jiang et al. converted discarded cigarette filters into an N, S-co-doped carbon nanofiber/carbon black (N, S-CNF/CB) composite, coating it onto GF separators. The polar N, S-CNF chemical absorbs NaPS while the nonpolar carbon black layer physically blocks their migration, with both layers maintaining rapid Na^+^ conductivity. As a result, Na–S cells with N, S-CNF/CB retain 527 mAh g^−1^ after 900 cycles at 0.5 C (0.035% fade per cycle) [102]. This biomass-derived strategy not only provides a cost-effective and sustainable route by repurposing waste materials but also features a relatively simple fabrication process, showing strong scalability and industrial feasibility for large-scale production. Similarly, biomass-derived carbons (from lychee seeds, coffee grounds, etc.) deliver hierarchical porosity and surface functionality for inherent polysulfide trapping and fast ion transport [103–105]. Extending this, Peng et al. fabricated an ultralight 3D N, S-co-doped cellulose aerogel (NSCA) and applied a 3 µm, 0.2 mg cm^−2^ coating. In symmetric cell CV, NSCA shows rapid polysulfide conversion kinetics. In full Na–S cells, it delivers 788.8 mAh g^−1^ at 0.1 C after 100 cycles, and with only 0.059% decay per cycle over 1000 cycles at 1 C (Fig. 4a-c) [106]. Leveraging a conventional papermaking process, the NSCA@GF separator achieves excellent scalability, low material cost, and compatibility with roll-to-roll continuous manufacturing, making it a promising candidate for future large-scale applications.

Overall, these interfacial engineering strategies, ranging from heteroatom-doped and biomass-derived carbons to ultralight aerogels, combine strong chemical binding, high conductivity, and tunable porosity to suppress polysulfide migration and establish the foundation for next-generation, high-energy, long-life sulfur-based batteries. From an economic perspective, the scalability of different interface engineering strategies remains an important consideration for practical M-S batteries. MOF- and COF-based separators offer exceptional tunability and precise ion-selective properties, but their relatively high synthesis cost and complex processing could hinder large-scale commercialization. In contrast, biomass-derived carbons, low-cost polymers, and naturally abundant inorganic fillers provide more feasible and sustainable pathways for industrial-scale applications, where balancing performance enhancement with cost-effectiveness will be critical.

##### Polar Metal Compound

Polar metal oxides possess unshared lone‐pair electrons that form strong chemical bonds with cations or polar polysulfide sites, markedly increasing adsorption energy and battery capacity [107–109]. When combined with other functional materials, metal oxides also help optimize interfacial structures, further stabilizing cycling behavior and enhancing reaction kinetics. For example, on the Al_2_O_3_-Nafion composite GF separator mentioned in the previous chapter, diffusion experiments and self-discharge tests confirmed that Nafion’s SO_3_H electrostatic repulsion and Al_2_O_3_ chemical adsorption synergistically slow NaPS shuttling [68]. Ye et al. designed a Janus separator combining a V_2_O_5_ microsphere catalytic layer that both traps and accelerates LiPS conversion and an Al_2_O_3_ nanosheet conductive layer to regulate ion flux and suppress dendrite growth, resulting in just 0.036% capacity decay over 2000 cycles [110]. Following the widespread application of metal nanoparticles in energy storage, Liu’s team developed core–shell MFe@FeO_x_ (M = Co, Ni, Mn) nanoparticles via solid-phase synthesis (Fig. 4d, e), in which the FeO_x_ shell anchors polysulfides and the conductive MFe core enhances conversion kinetics, enabling Na–S cells to retain 320 mAh g^−1^ after 1200 cycles with nearly 100% coulombic efficiency at 2 A g^−1^ [111]. Similarly, Zhang et al. anchored Fe_3_Se_4_ nanoparticles on 3D N-doped porous carbon nanosheets to suppress polysulfide shuttling and accelerate sulfur conversion, enabling RT Na–S cells to deliver 323 mAh g^−1^ at 2 A g^−1^ and retain 72% capacity after 500 cycles at 1.0 A g^−1^ [112]. In the recent study, a new type of composite material, Co-NCs/PW_10_V_2_, was explored as a modified separator in RT Na–S batteries, emphasizing its innovative role in capturing and immobilizing NaPS (Fig. 4f, g) [113]. The precisely defined V sites in PW_10_V_2_ enhance polysulfide adsorption (Fig. 4h) and enable multi-electron redox, functioning as a bidirectional molecular catalyst, while the Co-NCs scaffold improves conductivity and exposes catalytic sites. Furthermore, in situ electrochemical Raman spectroscopy confirmed that the [PW_10_V_2_O_40_]^5−^ polyoxoanions catalyze the reversible multi-electron conversion between low-order and high-order NaPS, thereby accelerating the overall redox kinetics (Fig. 4j). As a result, the modified separator delivers an initial discharge capacity of 1476.9 mAh g^−1^, retains 846.7 mAh g^−1^ at 5 C, and exhibits only 0.012% capacity decay per cycle over 4000 cycles (Fig. 4i). Under diverse disciplinary backgrounds, Zhang et al. applied an external acoustic field to excite the piezoelectric effect of polarized BaTiO_3_ nanoparticles (Fig. 4k) [114]. This achieved synergistic regulation of directional polysulfide anchoring and dynamic Na^+^ homogenization in Na–S batteries, offering an interdisciplinary dynamic solution to suppress the shuttle effect and dendrite growth (Fig. 4l). Although polar metal complexes remain unexplored in the separator design of K–S systems, analogous approaches have been reported in Mg-S cells [115].

Metal sulfides exhibit strong adsorption toward polysulfides and excellent electronic conductivity, and their intense interaction with M_2_S/M_2_S_x_ phases reduces reaction barriers, promotes M^+^ transport and regulates the deposition of insoluble sulfides, thereby accelerating surface-mediated redox reactions and improving overall electrochemical performance in sulfur-based batteries [116, 117]. Consequently, a variety of metal sulfides have been integrated into alkali M-S systems, with layered MoS_2_ receiving particular attention for its abundant active sites and high conductivity [118]. Xu et al. synthesized MoS_2_-decorated hollow carbon spheres (HCS/MoS_2_) that confine dissolved NaPS within their cores to enhance sulfur utilization and mitigate volume expansion, while the same material coated on a GF separator adsorbs escaped polysulfides at the interface to prevent diffusion to the anode (Fig. 4m) [119]. Visual adsorption tests and XPS analyses confirmed strong interactions between HCS/MoS_2_ and Na_2_S_6_. During discharge, in situ sodiation of MoS_2_ to Na_x_MoS_2_ (x < 2) generates additional anchoring sites. This dual-anchoring mechanism significantly improves capacity retention in Na–S batteries. Similarly, pyrite type FeS_2_, an abundant, low-cost, low-toxicity coal byproduct with robust stability, has emerged as a compelling choice. Huang et al. decorated separators with FeS_2_@C nanocubes and carbon nanotubes to form a conductive carbon-fiber network as the first defense and porous Fe_2_S cores as the second (Fig. 4n) [120]. The carbon framework ensures rapid ion/electron transport while the Fe_2_S cores bind and catalyze polysulfides (Fig. 4p). Time-resolved XRD studies further revealed that the sulfur conversion follows a stepwise pathway, demonstrating that the hierarchical polar hybrid structure not only provides strong polysulfide immobilization but also accelerates their reversible conversion (Fig. 4o). As a result, Na–S cells with a sulfur loading of 7.6 mg cm^−2^ deliver only 0.024% capacity decay per cycle over 1000 cycles at 2 C, demonstrating highly competitive performance.

##### Metal Ions and Organic Ligand Chelates

MOFs bind polysulfides via coordination of metal d-orbitals and S_x_^2−^ clusters, but their expensive organic linkers hinder large-scale use [52, 75]. As a lower-cost alternative, metal–organic chelates leverage Lewis acid–base interactions to immobilize polysulfides. For example, Jiang et al. synthesized Fe^3+^/polyacrylamide nanospheres (FPN) via scalable Fe^3+^-PAM coordination, then co-coated FPN and graphene onto GF separators to form a mesoporous interlayer that anchors polysulfides and ensures fast Na^+^ transport [121]. Graphene’s high surface area also prevents cracks in the otherwise brittle FPN layer. When paired with a carbonized N-doped carbon nanosphere cathode, the assembled Na–S battery achieved 639 mAh g^−1^ after 400 cycles at 0.1 C, along with significantly enhanced rate capability and cycling stability (Table 3).

**Table 3:** Electrochemical performance summary of various absorption interfaces for M-S batteries

Separator type	Battery type	thickness (material loading)	Sulfur loading (mg cm^−2^)	Practical capacity (mAh g^−1^) (Rate)	Cycling performance (mAh g^−1^) (Cycles/Rate)	Capacity decay (%)		References
SWCNT@PP layer	K–S	0.2 mg cm^−2^	1.0	1144 (0.1 C)	600 (600/0.1 C)	0.95		[94]
N, S-CNF/CB	Na–S	15 μm (0.16 mg cm^−2^)	0.6	1140 (0.1 C)	527 (900/0.5 C)	0.035		[102]
NSCA@GF	Na–S	3.0 μm (0.2 mg cm^−2^)	1.0–1.5	952.2 (0.5 C)	527 (1000/1 C)	0.059		[106]
White graphite@ PVDF-HFP/PBMA	Na–S	~ 207 μm	0.6–0.8	1034 (50 mA g^−1^)	499 (500/100 mA g^−1^)	0.048		[77]
CoFe@FeO_x_	Na–S	0.25–0.4 mg cm^−2^	1.5	772 (0.2 A g^−1^)	320 (1200/2 A g^−1^)	0.042		[111]
CoNCs/PW_10_V_2_	Na–S	14.5 μm	1.5	1476.9 (0.5 C)	429.4 (4000/10 C)	0.012		[113]
HCS/MoS_2_	Na–S	10 µm	~ 3.5	1309 (0.1 C)	559 (1000/1 C)	0.049		[119]
Janus-type LCL-TCL	Na–S	1.0 mg cm^−2^ on each side	1.5	1050 (0.5 C)	835 (1000/1 C)	0.013		[120]
FPNs-G	Na–S	10 µm	0.68	732 (0.5 C)	396 (800/0.5 C)	0.040		[121]
LCL-TCL	Li–S	1.0 mg cm^−2^ on each side	~ 2.0	1050 (0.5 C)	≈400 (2000/1 C)	0.036		[110]

Achieving optimal polysulfide adsorption requires a balance: it must be strong enough to suppress shuttle and improve coulombic efficiency, yet moderate enough to allow facile polysulfide cleavage and reconversion for high active material utilization. In addition, scaling up alkali M-S batteries demands cost-effective, eco-friendly materials and processes to ensure both economic viability and sustainability.

#### Targeted Acceleration Channel: Catalytic Functional Interface

Catalysis in batteries, much like an airport fast track, speeds up molecular transformations without being consumed, while adsorption anchors reactants to active sites and enhances catalyst-substrate interactions. These two processes reinforce each other because catalytic sites promote stronger adsorption and anchored species undergo more efficient conversion. Techniques such as heterogeneous junctions, defect engineering and single‑atom catalysts further amplify this synergy [122–124]. By integrating both adsorptive and catalytic functions at the interface, alkali M-S batteries lower reaction energy barriers, trap long‑chain polysulfides and accelerate their conversion into shorter species, thereby suppressing shuttle effects and enabling highly reversible cycling.

##### Material Customization Strategy

**Fig. 5:**
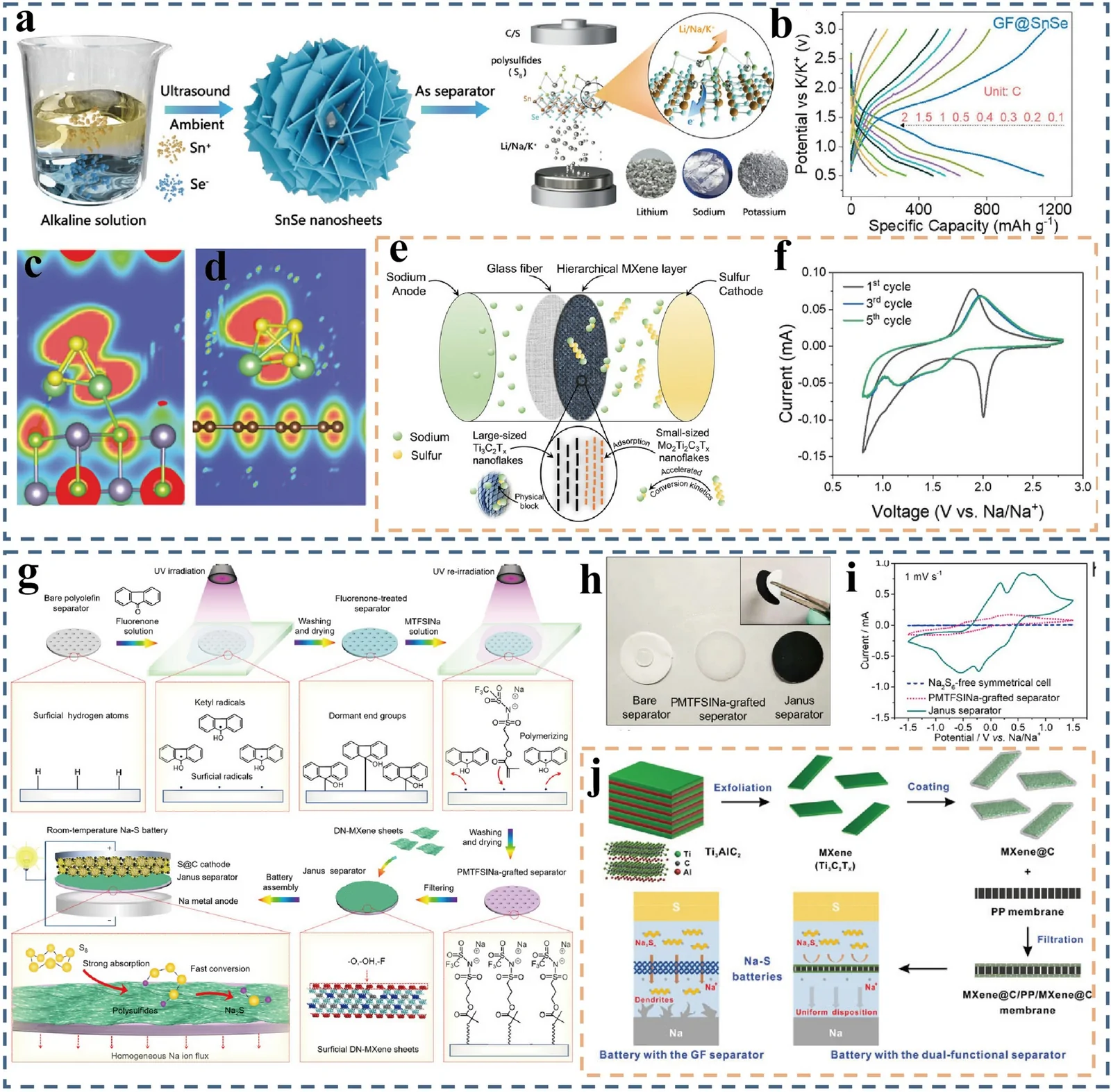
**a** Synthetic procedure of SnSe nanosheets and their incorporation in M-S batteries. **b** Galvanostatic charge/discharge profiles of the K–S coin cells based on the GF@SnSe separators. Band-decomposed charge density distribution of the **c** SnSe-Li_2_S_4_ adsorption system; **d** graphene-Li_2_S_4_ adsorption system. Reproduced with permission [127]. Copyright 2024, Wiley–VCH.** e** Configuration of the RT Na–S battery system with GF@Hybrid MXene. **f** CV profiles. Reproduced with permission [130]. Copyright 2023, Wiley–VCH.** g** Preparation of PMTFSINa-grafted DN-MXene-coated Janus separators for RT Na–S batteries. **h** Optical images of different separators. **i** CV curves of symmetrical cells. Reproduced with permission [131]. Copyright 2021, Wiley–VCH. **j** Preparation of the MXene@C nanosheets and MXene@C/PP/MXene@C dual-functional separator. Reproduced with permission [132]. Copyright 2022, Wiley–VCH

MoSe_2_ has shown significant catalytic activity in Li–S batteries and was recently applied to Na–S systems [125]. Jiang et al. introduced a N-doped hollow carbon sphere interlayer coated with 2H-phase MoSe_2_ nanoparticles and graphene oxide (2H-MoSe_2_/H-HCS/GO) [126]. This layer immobilizes NaPS intermediates via strong chemical adsorption and accelerates redox kinetics, improving sulfur utilization. PDOS analysis and DFT calculations indicate that MoSe_2_ surfaces exhibit favorable Na_2_S activation and ultralow Na^+^ diffusion barriers. As a result, Na–S batteries with a high sulfur content of 71.4 *wt*% deliver 99.6% coulombic efficiency and only 0.077% capacity decay per cycle over 500 cycles at 0.5 C. Meanwhile, a multifunctional separator based on SnSe nanosheets has likewise been shown to strengthen chemical anchoring and catalytic conversion of polysulfides in Li/Na/K–S cells (Fig. 5a, b) [127]. Charge density distribution of the SnSe-Li_2_S_4_ complex confirms effective electronic pathways between SnSe and adsorbed polysulfides (Fig. 5c, d). Moreover, the SnSe layer enhances K_2_S conversion kinetics in K–S batteries, as evidenced by sharply defined redox peaks. The versatility of these interfacial designs across all three alkali M-S systems represents a major advance in separator engineering for next-generation M-S batteries.

MXenes, a family of 2D materials, combine high electrical conductivity, abundant surface functional groups, structural tunability and intrinsic catalytic activity to anchor polysulfides, accelerate sulfur redox and suppress shuttle effects in alkali M-S batteries [128, 129]. Wang and co-workers designed a multifunctional dual-layer MXene modified separator by selectively etching large-sized Ti_3_C_2_T_x_ and small-sized Mo_2_Ti_2_C_3_T_x_ MXenes using different acids (Fig. 5e) [130]. In this structure, the inner layer of large Ti_2_C_3_T_x_ nanosheets forms a dense physical barrier that inhibits polysulfide diffusion and prevents short circuits. The outer layer, composed of smaller Mo_2_Ti_2_C_3_T_x_ nanosheets, provides a high surface area and abundant Mo-rich catalytic sites to accelerate polysulfide conversion and retain active species near the cathode. Notably, CV analysis (Fig. 5f) reveals a significant decrease in the reduction peak of long-chain NaPS after only three cycles. This suggests a rapid catalytic transformation into insoluble short-chain species, which enhances redox kinetics in the Na–S battery.

The thick structure (~ 300 μm) and large pore size of GF separators hinder energy density improvement and increase short circuit risk due to dendrite penetration. Their weak mechanical strength also limits scalability. In contrast, modified polyolefin separators offer better electrolyte wettability and mechanical robustness. In a related study, the same group proposed a controlled modification strategy for commercial polyolefin separators via a “grafting-filtration” method, resulting in a Janus membrane functionalized with PMTFSINa and nitrogen-rich defect-engineered MXene (DN-MXene) [131]. The single-ion-conductive polymer interface significantly improves electrolyte wettability, reduces electrolyte consumption, and concurrently mitigates polysulfide diffusion and Na dendrite growth. Additionally, the DN-MXene layer functions as an electrocatalyst, promoting the conversion of NaPS to Na_2_S and further enhancing internal reaction kinetics. This synergistic interfacial design substantially improves the electrochemical performance of RT Na–S batteries (Fig. 5g-i). Beyond energy storage, such advanced separator modification strategies may also open new avenues for applications in seawater desalination, wastewater treatment, and osmotic energy conversion, highlighting their broad technological potential. Additionally, Fang et al. fabricated an ultrathin (1.5 µm) binder-free MXene@C coating on PP separators via vacuum filtration. This coating achieved strong adhesion, improved Na^+^ flux uniformity, and effective dendrite suppression [132]. The assembled Na–S batteries retained 928 mAh g^−1^ after 650 cycles at 0.5 C with 95.8% capacity retention (Fig. 5j).

##### Doping-Defect Engineering

**Fig. 6:**
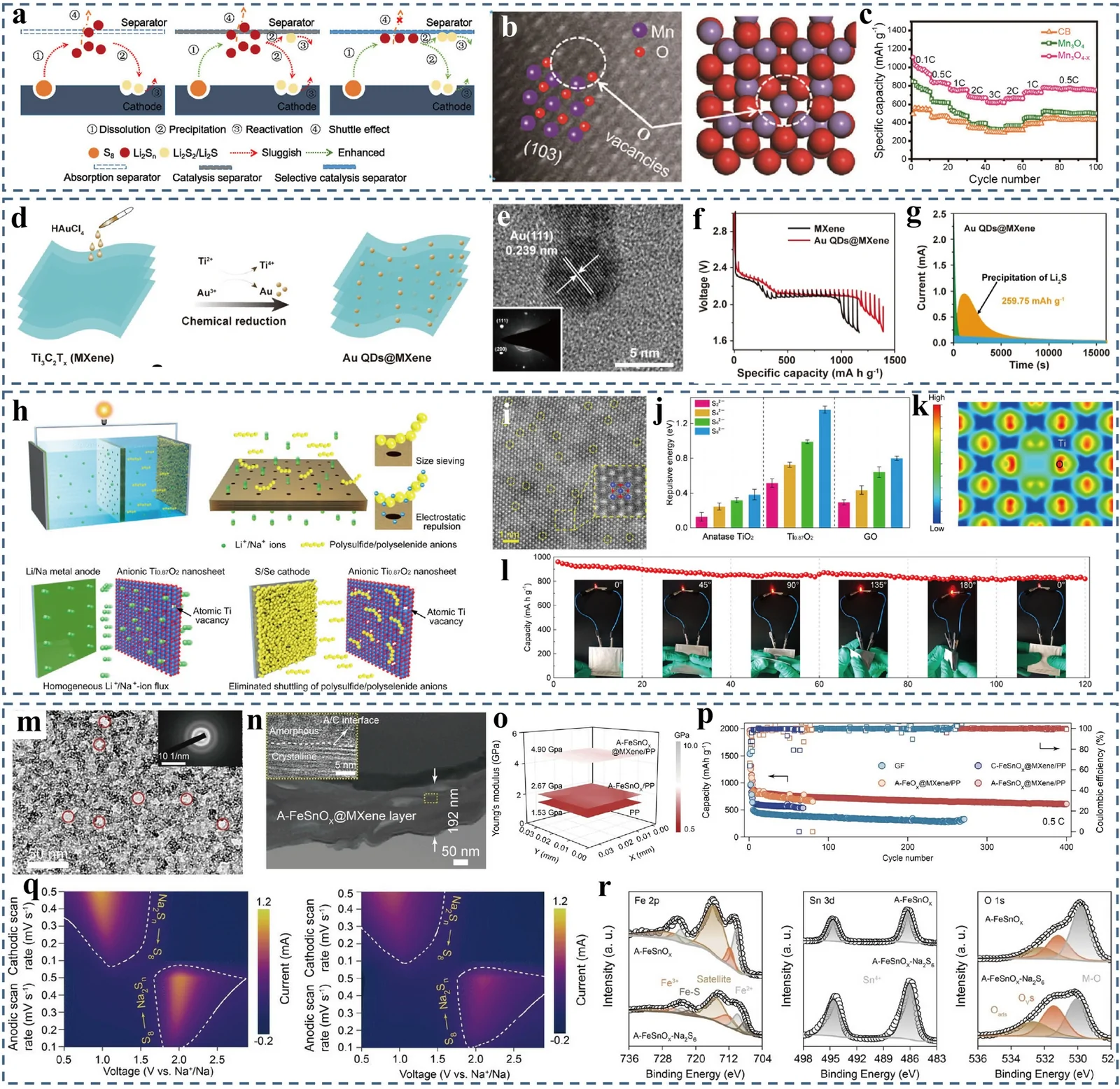
**a** Schematic illustration to tackle the heterogeneous reaction and the “shuttle effect” in Li–S batteries. **b** Structure model of Mn_3_O_4−x_. **c** Rate performance. Reproduced under terms of the CC-BY license [133]. Copyright 2023, The Authors, published by Wiley. **d** Preparation scheme and **e** HRTEM images of Au QDs@MXene heterostructure. **f** GITT curves. **g** Li_2_S deposition profile. Reproduced under terms of the CC-BY license [134]. Copyright 2024, The Authors, published by OAE Publishing Inc.** h** Schematic illustration of Ti_0.87_O_2_/PP separator. **i** HAADF-STEM image. **j** Repulsion energies of various polysulfide S_x_^2−^ on different materials. **k** Charge density plot. **l** Cycling performance of the Li–S pouch cell. Reproduced under terms of the CC-BY license [135]. Copyright 2021, The Authors, published by Springer Nature.** m** TEM images. **n** Cross-sectional TEM image of the A-FeSnO_x_@MXene/PP separator. **o** Young’s modulus 3D contour maps of different separators. **p** Cycling performance. **q, r** Polysulfides adsorption and catalytic capability of the A-FeSnO_x_@MXene/PP Separator. Reproduced with permission [136]. Copyright 2024, Wiley–VCH

Defect engineering plays a dual role in alkali M-S batteries: it suppresses polysulfide dissolution and diffusion via strong chemisorption at defect sites, while also enhancing liquid-phase conversion kinetics through defect-induced electronic modulation. Zhu et al. developed an oxygen-vacancy-rich Mn_3_O_4-x_ catalyst, where abundant vacancies not only act as active sites for anchoring polysulfides but also lower energy barriers for their conversion into insoluble Li_2_S_2_/Li_2_S (Fig. 6a, b) [133]. This synergistic effect enables the Li–S battery to achieve an ultralow capacity decay of 0.028% per cycle over 2000 cycles at 2.5 C, even under high sulfur loading (Fig. 6c). Combined experimental and theoretical analyses further reveal a clear correlation between defect concentration and catalytic performance, providing valuable guidance for the rational design of advanced catalysts.

Defect engineering has emerged as a powerful tool to mitigate the polysulfide shuttle in Li–S batteries and has been extended to Na–S systems to boost electrocatalytic activity and suppress NaPS. For example, a recent study constructed Au quantum dots (Au QDs) anchored on MXene separators (Au QDs@MXene) (Fig. 6d, e) [134]. MXene’s high conductivity (up to 10^4^ S cm^−1^) and polar -O/-OH groups chemisorb polysulfides, while uniformly dispersed ∼6 nm Au QDs accelerate Li_2_S_2_/Li_2_S conversion via quantum confinement effects and abundant active sites. Furthermore, UV–vis and XPS analyses confirm its efficient polysulfide anchoring capability, while GITT and Li_2_S precipitation experiments elucidate the rapid charge transfer properties (Fig. 6f, g). Building on this, Wang’s group coated PP separators with Ti_0.87_O_2_ nanosheets containing abundant Ti vacancies, which repel polysulfides and facilitate Li^+^ transport [135]. This design enables flexible Li–S pouch cells to maintain stable cycling even under bending conditions (Fig. 6h-l). This work highlights the potential of atomic-scale ion regulation in advancing the performance of alkali metal-S/Se batteries, offering valuable insights for the development of next-generation energy storage systems. In Na–S systems, Zhao et al. designed amorphous 2D FeSnO_x_ nanosheets rich in oxygen vacancies and nanopores, and coated them together with a small amount of MXene onto a PP separator (Fig. 6m, n) [136]. The vacancies provide strong polysulfide adsorption and catalytic activity, while the nanopores facilitate uniform Na^+^ transport and deposition. CV curves and XPS results confirm the significantly improved catalytic performance, underscoring the essential role of oxygen vacancies in chemically anchoring and converting polysulfides (Fig. 6q, r). In addition, the MXene-derived amorphous/crystalline interface, with a Young’s modulus of 4.9 GPa, reinforces the brittle oxide layer and suppresses dendrite growth (Fig. 6o). As a result, RT Na–S batteries deliver 610.3 mAh g^−1^ after 400 cycles at 0.5 C, with a high coulombic efficiency of 99.9% (Fig. 6p). Notably, unlike past studies on crystalline defects, this work leverages amorphous disorder. DFT results reveal that Sn incorporation lowers vacancy formation energy, increases Fe^2+^ concentration, and reduces Na^+^ migration barriers to approximately 0.11 eV, thereby enhancing catalytic performance. Besides, Yang and Zhang further developed Fe_1.88_C_0.12_@CNT electrocatalysts with carbon and Fe vacancies that anchor and convert polysulfides, delivering exceptional cycling stability and capacity retention in Na–S cells (Table 4) [137]. In addition, catalytic and mediated strategies have been shown to effectively accelerate sulfur redox kinetics. Organopolysulfurs act as internal redox co-mediators through S–S exchange, lowering activation barriers and enhancing polysulfide conversion. Meanwhile, chalcogen doping (e.g., Se/Te), solvation/anion regulation, and interfacial engineering further promote uniform Li_2_S nucleation and improve charge transfer, providing a complementary pathway to boost reaction kinetics and suppress polysulfide shuttle [138]. Despite these advances, scalable, controllable defect fabrication and long-term defect stability remain critical challenges for practical alkali M-S batteries. Recent studies, combining experiments and DFT, show that the interaction strength between alkali cations and defect sites depends strongly on ionic radius, solvation, and polarizability. Li^+^ features strong solvation that effectively screens its interaction with defect sites, yielding moderate binding of Li_2_S_x_ on many defect-rich oxides [139]. In contrast, the weaker solvation and higher polarizability of Na^+^/K^+^ generally increase the effective binding of Na_2_S_x_/K_2_S_x_ on defect sites, which helps immobilize polysulfides but may cause over-adsorption when vacancy densities are excessive, thereby blocking active sites and slowing conversion. Hence, optimizing defect density and tuning local electronic structure are essential to balance anchoring and kinetics [140, 141].

**Table 4:** Electrochemical performance summary of various catalytic interfaces for M-S batteries

Separator type	Battery type	Thickness (material loading)	Sulfur loading (mg cm^−2^)	Practical capacity (mAh g^−1^) (Rate)	Cycling performance v(mAh g^−1^) (Cycles/Rate)	Capacity decay (%)	References
2H-MoSe_2_/N-HCS/GO	Na–S	0.56 mg cm^−2^	0.64	1945 (0.5 C)	484 (500/0.5 C)	0.077	[126]
GF@SnSe	Li–S Na–S K–S	20 µm	1.0	1529 (0.1 C) 1127 (0.1 A g^−1^) 1035 (0.1 C)	425.9 (800/3 C) 479 (1000/0.1 A g^−1^) 436 (180/0.2 C)	0.049	[127]
GF@Hybrid MXene	Na–S	200 nm	1.0	562 (1 C)	357 (500/1 C)	0.073	[130]
PMTFSINa/DN-MXene	Na–S	NA	NA	1644 (0.1 C)	646 (500/0.5 C)	0.10	[131]
Ti_3_C_2_T_x_/Mo_2_Ti_2_C_3_T_x_	Na–S	200 nm	1.0	992 (0.1 C)	357 (500/1 C)	0.073	[132]
A-FeSnO_x_@MXene	Na–S	200 nm (0.1 mg cm^−2^)	NA	1409.6 (0.1 C)	610.3 (400/0.5 C)	0.073	[136]
2H-MoSe_2_/N-HCS/GO	Na–S	0.56 mg cm^−2^	0.64	530 (2 C)	484 (500/0.5 C)	0.077	[137]
Mn_3_O_4−x_ catalyst	Li–S	14 μm (0.19 mg cm^−2^)	NA	764 (0.1 C)	246 (2000/2.5 C)	0.028	[133]
Au QDs@MXene	Li–S	Au QDs 7 μm (0.032 mg cm^−2^)	2.5–5.1	1500 (0.2 C)	1065 (300/1 C)	0.097	[134]
Ti_0.87_O_2_/PP	Li–S	1.5 mg cm^−2^	NA	960 (0.2 C)	585 (5000/1 C)	0.0036	[135]

Recent advances in catalytic interface engineering highlight the importance of tailoring electronic structures to accelerate polysulfide redox kinetics and suppress shuttle effects across M-S systems. A recent review emphasizes that regulating defect chemistry, active sites, and orbital hybridization enables simultaneous enhancement of adsorption strength and catalytic conversion efficiency [142]. In addition, Schottky-type oxygen vacancies are generally associated with lattice anion deficiencies that alter the local crystal-field environment and can modulate the electronic band structure, potentially influencing the d-band center and electron distribution at catalytic interfaces. Sun et al. designed S-Co single-atom catalysts on N, S-doped carbon (S-Co-SACs/NSC), where sulfur coordination around Co tunes the d-band center and reinforces Co-S orbital coupling [143]. This strategy accelerates polysulfide conversion and reduces Li_2_S decomposition energy, achieving 834 mAh g^−1^ at 5 C and 7.0 mAh cm^−2^ at 7.8 mg cm^−2^ sulfur loading. Similarly, Dong et al. introduced a P/S co-coordination strategy that creates asymmetric electronic environments around metal centers, optimizing charge transfer and promoting uniform Li_2_S deposition for ultrahigh rate capability and long-term durability [144]. Extending these insights to K–S batteries, Song et al. developed a bifunctional catalytic interface by integrating tungsten single atoms (WSA) with W_2_C nanocrystals [40]. Guided by DFT calculations, WSA facilitates KPS migration through optimized adsorption energy, while W_2_C accelerates the conversion kinetics of intermediate species, effectively preventing K_2_S/K_2_S_2_ accumulation and catalyst deactivation. This synergistic catalytic design enables a high reversible capacity of 1504 mAh g^−1^ and outstanding cycling stability. Collectively, these studies demonstrate that combining orbital hybridization engineering, asymmetric coordination environments, and bifunctional catalytic architectures provides a versatile framework for regulating interfacial reactions and unlocking the potential of Li/Na/K–S batteries.

In summary, ion-selective interfaces provide efficient shuttle suppression with relatively low design complexity, adsorption-based interfaces achieve stronger polysulfide immobilization, and catalytic interfaces enhance redox kinetics by accelerating intermediate conversion. In practice, hybrid designs that integrate these mechanisms are increasingly adopted, offering a balanced regulation of ion selectivity, adsorption strength, and catalytic activity. Such synergistic approaches hold great promise for enabling next-generation high-performance Li/Na/K–S batteries. Although Li/Na/K–S batteries share similar sulfur redox pathways, differences in alkali metal properties lead to distinct challenges in separator interface design. In Li/Na–S systems, the main focus is on mitigating the polysulfide shuttle effect through physical confinement and catalytic conversion to accelerate redox kinetics. K–S batteries, still in early development, face more severe end-product accumulation due to the poor reversibility of K_2_S. Therefore, separator interfaces should emphasize the catalytic promotion of complete conversion and reversible oxidation of low-valent KPS. Rationally tailoring these interfaces to address system-specific issues is essential for unlocking the full electrochemical potential of each battery type.

### Interlayer Interface Modification

In alkali M-S batteries, introducing a functional interlayer is a key interface engineering strategy to suppress polysulfide migration and alleviate the shuttle effect. These interlayers, whether artificially deposited or formed in situ, act as physical barriers while also enhancing redox kinetics, improving sulfur utilization, and buffering volume changes during cycling. On the anode side, they help regulate ion flux and suppress dendrite growth due to their mechanical robustness and flexibility. Achieving these multifunctional effects requires interlayers with selective permeability, strong adsorption capacity, catalytic activity, and mechanical robustness, which are vital for ensuring high efficiency and long-term stability in these battery systems.

#### Cathode Side Interface

**Fig. 7:**
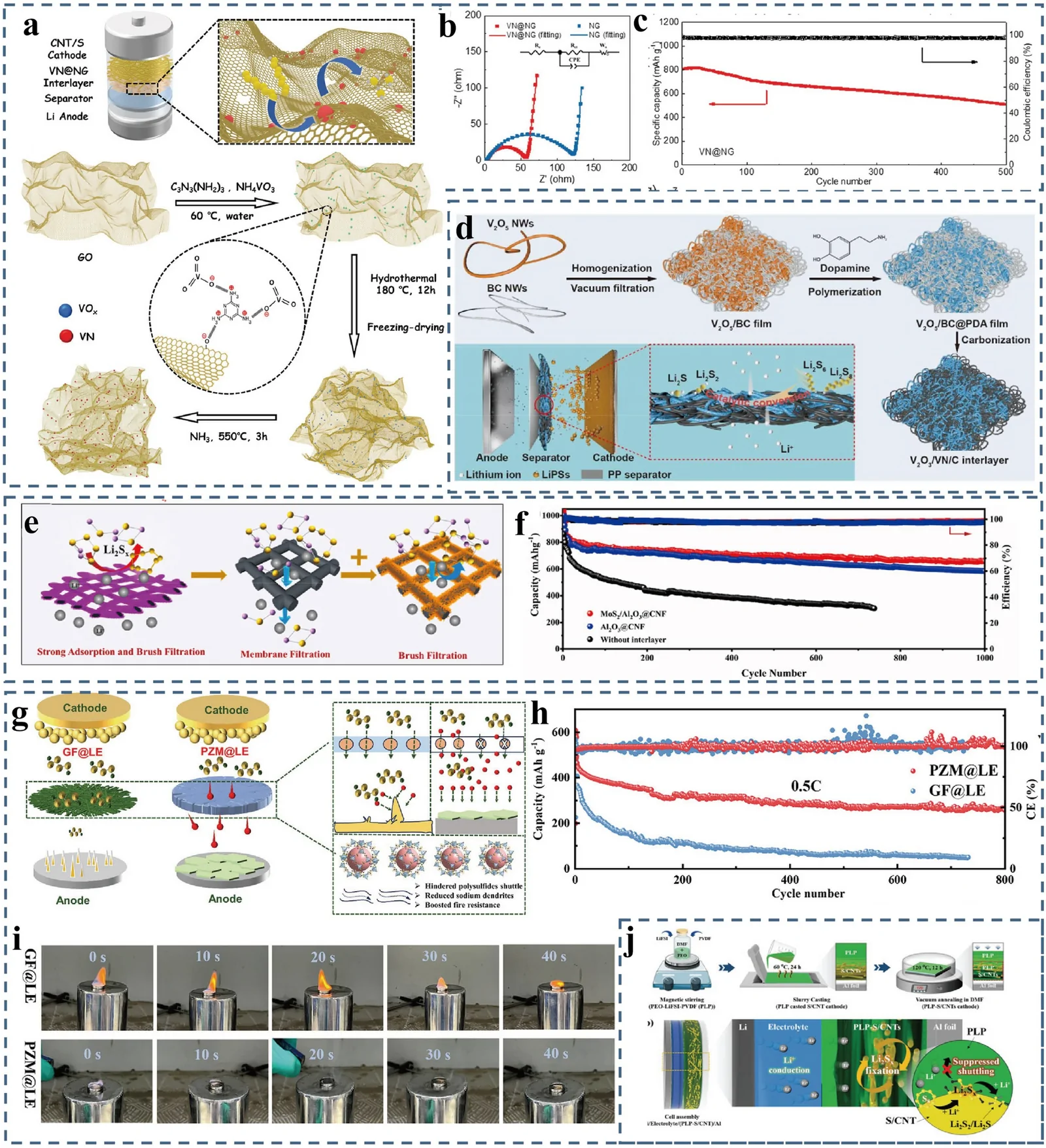
**a** Preparation of the VN@NG. **b** EIS plots. **c** cycling stability. Reproduced with permission [145]. Copyright 2021, Wiley–VCH. **d** Fabrication process and mechanism of V_2_O_3_/VN/C interlayer. Reproduced with permission [146]. Copyright 2022, Elsevier. **e** Strong adsorption and enhanced brush filtration effect of the fabricated interlayer. **f** Long-term cycling performance. Reproduced with permission [147]. Copyright 2022, Elsevier. **g** Graphical sketch of PZM membrane. **h** Long-term cycling performance. **i** Flame-retardant tests with GF@LE and PZM@LE at different durations. Reproduced with permission [78]. Copyright 2024, Wiley–VCH. **j** Preparation of the PLP-S/CNTs cathode. Reproduced with permission [152]. Copyright 2023, Wiley–VCH

Similar to the functionalized separator, the interlayer also requires the characteristics of hindrance-absorption-conversion. Taking Li–S batteries as an example, Xia et al. loaded VN nanoparticles into porous N-doped graphene (VN@NG), effectively reducing the activation energy of Li_2_S_4_ conversion, with capacity attenuation of only 0.075% after 500 cycles at 2 C (Fig. 7a-c) [145]. Subsequently, the interlayer composed of V_2_O_3_/VN and the flexible conductive carbonized bacterial cellulose (CBC) further reduced the capacity attenuation rate to 0.035% after 1000 cycles (Fig. 7d) [146]. Wu et al. co-deposited MoS_2_ and Al_2_O_3_ on carbon nanofibers (MoS_2_/Al_2_O_3_@CNF) by magnetron sputtering. By taking advantage of the increased specific surface area and active sites, the assembled battery achieved a low attenuation of 0.035% after 1000 cycles at 0.5 C (Fig. 7e, f) [147]. These designs enhance the adsorption and transformation of polysulfides from different perspectives, effectively suppressing the shuttle effect. In Na–S systems, V_2_O_3_ nanoparticles grown in one step on electrospun carbon nanofibers (V_2_O_3_@CNF) form a conductive network that accelerates Na^+^ transport and anchors polysulfides, significantly improving cycling stability [148]. Xu and Qi’s team prepared a porous ZIF-derived membrane (PZM) with hierarchical channels that block polysulfide shuttling and inhibit dendrite growth (Fig. 7g, h). Its nonflammable, low-electrolyte design further enhances safety and cost-effectiveness (Fig. 7i), enabling stable performance over 800 cycles at 0.5 C [78]. Incorporating tetrachlorobenzoquinone into a polyphenylene sulfide solid separator (PPS-SSS) also delivers efficient polysulfide adsorption and shuttle suppression [149]. Extending this interlayer strategy to K–S batteries, Sharma’s group fabricated a self-supporting CBC layer whose porous nanofiber network ensures uniform sulfur distribution, captures KPS under high sulfur loading and suppresses dendrite growth, thereby achieving long cycle life and excellent capacity retention at high current density [150]. Based on this, they introduced banana fibers in situ to modify the carbonized bacterial cellulose, preparing a self-supporting and binder-free cathode, while continuing to use CBC as the interlayer. The cycle stability of the K–S battery has been further enhanced [151].

Surface coating technology has the advantages of strong versatility, simple processing steps and controllable costs, and is often used for battery interface decoration. Directly applying a conformal layer on the cathode can buffer the mechanical stress caused by the large volume change of sulfur and also inhibit the dissolution of polysulfides to reduce the shuttle effect. For example, Il-Doo Kim and co-workers employed a simple coating followed by controlled thermal annealing to infuse a PEO/LiFSI/PVDF (PLP) gel polymer electrolyte into an S/CNT scaffold (Fig. 7j), creating a composite cathode with an ultrathin protective shell [152]. The PLP layer adheres tightly to the S/CNT network and minimizes the generation of soluble polysulfide intermediates, thereby driving as much active sulfur as possible toward final conversion. DFT calculations show that LiFSI preferentially interacts with PEO with a high donor number (DN) to suppress the solubility of polysulfides, while PVDF with a low DN encapsulates sulfur in situ, promoting its direct conversion to Li_2_S_2_/Li_2_S. This dual-mechanism coating combines DN tuning with simple surface deposition, synergistically inhibiting the dissolution and shuttling of polysulfides. As a result, the PLP-coated Li–S battery delivers 573.6 mAh g^−1^ at 0.5 C over 1000 cycles and 318.1 mAh g^−1^ at 6 C. This performance significantly narrows the gap between laboratory results and industrial viability for Li–S batteries (Table 5).

**Table 5:** Electrochemical performance summary of various cathode-side interlayers for M-S batteries

Separator type	Battery type	thickness (material loading)	Sulfur loading (mg cm^−2^)	Practical capacity (mAh g^−1^) (Rate)	Cycling performance (mAh g^−1^) (Cycles/Rate)	Capacity decay (%)	References
VN@NG	Li–S	13 µm (0.25 mg cm^−2^)	1.0	830 (0.1 C)	509 (500/2 C)	0.075	[145]
V_2_O_3_/VN/C	Li–S	15 μm	1.0	1419.5 (0.1 C)	768.7 (1000/1 C)	0.037	[146]
MoS_2_/Al_2_O_3_@CNF	Li–S	23 μm	4.05	1200.5 (0.2 C)	653.91 (1000/0.5 C)	0.035	[147]
PEO/LiFSI/PVDF (PLP)	Li–S	9.0 nm (0.15 mg cm^−2^)	1.8–2.1	318.1 (6 C)	573.6 (1000/0.5 C)	0.039	[152]
V_2_O_3_@CNF	Na–S	3.0 mg cm^−2^	0.5–0.7	1162 (0.1 C)	95 (1000/2 C)	0.076	[148]
PZM	Na–S	1.0 mm	NA	753 (0.1 C)	≈527 (800/0.5 C)	0.038	[78]
CBC	K–S	NA	2.0	263 (0.5 C)	73 (500/2 C)	0.088	[150]

Compared with Li–S batteries, Na–S and K–S systems suffer even greater volume changes and polysulfide shuttling, yet electrode‐coating studies in these chemistries remain sparse despite their high research value and application potential. An ideal cathode coating needs to satisfy three criteria: (1) provide excellent electrical conductivity to accelerate electron/ion transport at the interface; (2) offer moderate polysulfide adsorption that confines soluble intermediates without impeding their conversion; (3) supply sufficient free volume to buffer the electrode’s volume expansion during cycling. Future work should thus aim to construct multifunctional composite layers that combine conductive networks, tailored adsorption sites and tunable porosity to synergistically suppress the shuttle effect, stabilize the electrode structure and extend cycle life in Na–S and K–S batteries.

#### Anode Side Interface

Na–S and K–S batteries face unique anode-interface challenges due to the inherent properties of Na and K metals. Their higher redox potentials render SEI components less stable in organic electrolytes, while the larger ionic radii amplify mechanical stress during volume changes, making the SEI prone to cracking. Moreover, the low melting points of Na/K and the high reactivity of K further destabilize the interface and accelerate electrolyte decomposition (Table 1). These combined issues elevate overpotentials, reduce reversibility, and erode the sulfur cathode’s energy density advantage. Since SEI formation is fundamentally a thermodynamic reduction of the electrolyte on the metal surface, and Na/K are more reactive than Li, engineering of the anode side interface is critical. Functional interlayers can block polysulfides, guide uniform metal deposition, and promote SEI self‐healing [153, 154]. Among them, artificial SEIs are constructed via electrolyte additives or surface coatings. Future work should integrate SEI chemistry with tailored interlayer architectures to deliver robust, long‐cycling alkali M-S batteries (Table 6).

**Table 6:** Electrochemical performance summary of various anode-side interlayers for M-S batteries

Separator type	Battery type	layer thickness (material loading)	Sulfur loading (mg cm^−2^)	Practical capacity (mAh g^−1^) (Rate)	Cycling performance (mAh g^−1^) (Cycles/Rate)	Capacity decay (%)	References
Sn(OTf)_2_ additive	Na–S	SEI ~ 10 nm	NA	1199 (0.5 A g^−1^)	906 (100/0.5 A g^−1^)	0.244	[155]
Li_x_SiS_y_/Nafion	Li–S	Li_x_SiS_y_: 200 nm Li-Nafion: 1.8 μm Total: 2.0 μm	1.0	1155 (0.1 C)	783 (300/0.5 C)	0.085	[156]
LN	Li–S	NA	1.2	1020 (0.5 C)	791.5 (250/0.5 C)	0.090	[157]
LiTe_3_ additive	Li–S	Concentration:0.1 M	2.5–4.5	459 (1 C)	350 (100/0.2 C)	0.178	[158]

##### Electrolyte Additives

**Fig. 8:**
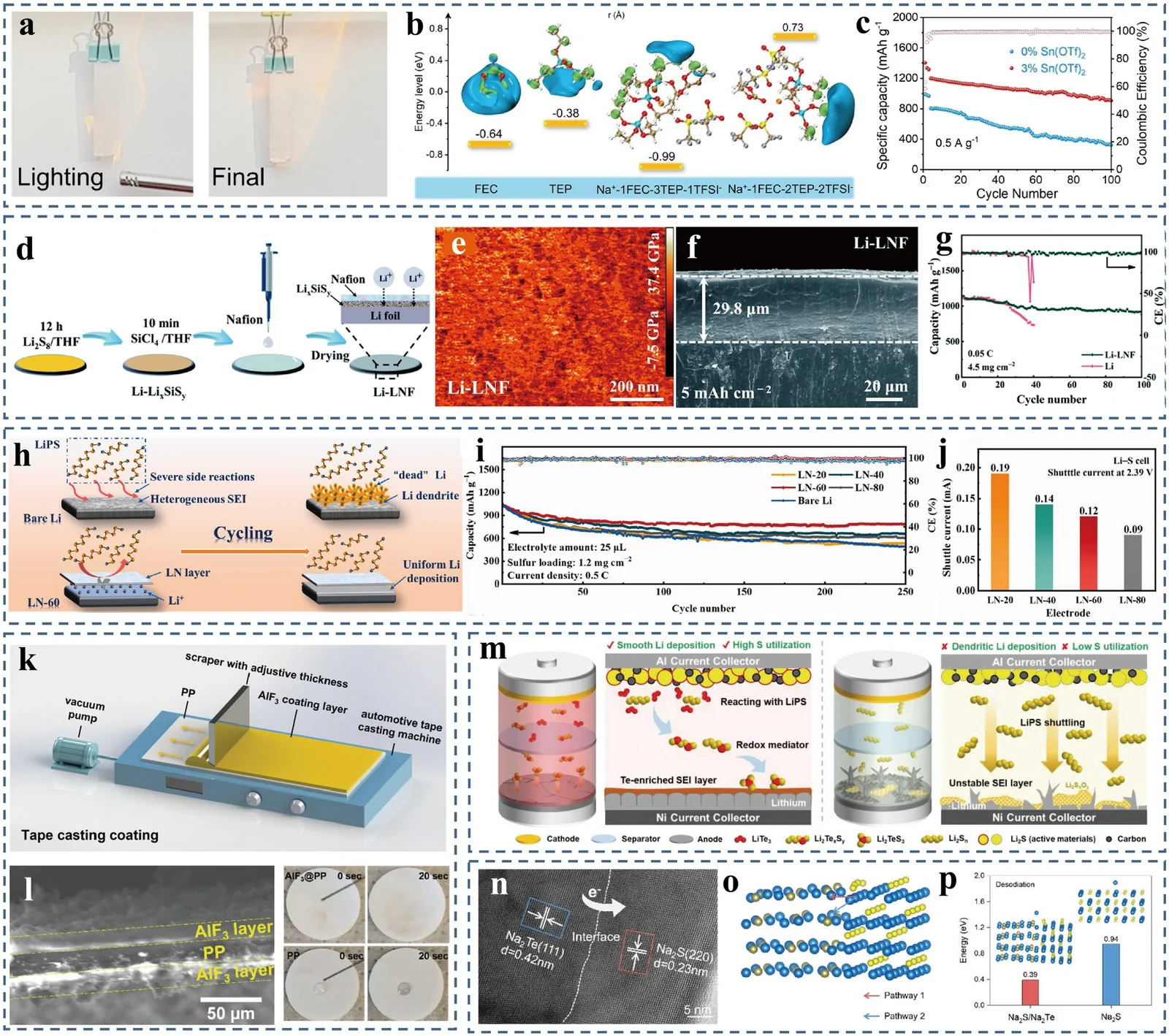
**a** Digital photos of the burning process. **b** LUMO energy level. **c**Cycling performance with and without Sn(OTf)_2_ additive. Reproduced with permission [155]. Copyright 2024, Wiley–VCH. **d** The fabrication process and **e** Young's modulus mappings of a Li-LNF electrode. **f** Cross-sectional SEM images of plated Li on the Li-LNF electrode. **g** Cycling performances. Reproduced under terms of the CC-BY license [156]. Copyright 2020, The Authors, published by the Royal Society of Chemistry. **h** Effect of the LN layer on shielding LiPS and uniform Li deposition. **i** Cycling performance. **j** Shuttle currents of Li–S cells for different anodes. Reproduced with permission [157]. Copyright 2021, Elsevier.** k** Schematic images and **l** SEM image of AlF3@PP separator. Reproduced with permission [159]. Copyright 2022, Wiley–VCH.** m** Anode-free Li–S cells with and without LiTe_3_ additive. Reproduced with permission [158]. Copyright 2023, Wiley–VCH. **n** HRTEM image of Na_2_S/Na_2_Te@C. **o** Potential Na migration pathways within the Na_2_S/Na_2_Te structure. **P** Formation energies necessary for Na extraction in the desodiation reactions of Na_2_S and Na_2_S/Na_2_Te. Reproduced with permission [160]. Copyright 2024, Wiley–VCH

FEC in situ generates a crystalline, inorganic-rich SEI on Na metal that both passivates the anode and promotes uniform Na^+^ migration, enhancing rate performance in Na/K systems. However, its ability to suppress polysulfide dissolution and shuttling remains limited [26]. Building on this, Yu et al. added Sn(OTf)_2_ to a flame-retardant phosphate electrolyte (Fig. 8a), forming a Na_15_Sn_4_-rich SEI on the anode and a Sn/NaSn CEI on the cathode that jointly suppress polysulfide shuttling and boost Na–S capacity from 322 to 906 mAh g^−1^ over 100 cycles at 0.5 A g^−1^ (Fig. 8b, c) [155]. Jin et al. developed a dual-layer SEI of Li_x_SiS_y_ and Nafion that combines mechanical robustness and Li^+^ conductivity with electrostatic polysulfide repulsion (Fig. 8d-g), delivering 783 mAh g^−1^ after 300 cycles at 0.5 C, and later simplified it to a single lithiated-Nafion coating that maintained 791.5 mAh g^−1^ over 250 cycles at 0.2 C [156, 157]. These studies underscore the versatility of artificial SEI strategies, including crystalline inorganic films, alloy-rich layers, polymer membranes and Nafion coatings, in stabilizing alkali metal anodes, mitigating shuttle effects and suppressing dendrites (Fig. 8h-j). Artificial SEI layers to date have largely focused on uniform metal ion deposition to suppress dendrites, yet alleviating shuttle effects and combining stable plating with enhanced redox kinetics remain a challenge. Introducing tellurium-based compounds has provided breakthroughs in both cathode and anode interface regulation. In Li–S batteries, Manthiram’s team used lithium tritelluride (LiTe_3_) as an electrolyte additive [158]. On the cathode, LiTe_3_ reacts with LiPS to form polytellurosulfides that serve as redox mediators, reducing the Li_2_S activation barrier and lowering the charging plateau from 3.3 V to 2.5 V while promoting uniform sulfur deposition. On the anode, migrating tellurium-sulfur species create a Li_2_TeS_3_/Li_2_Te-rich SEI with 50% lower impedance, guiding homogeneous Li plating and enabling anode-free cells to retain 70% capacity over 60 cycles under lean electrolyte conditions (E/S ratio = 5) (Fig. 8m).

##### Surface Coatings

Protective layers are traditionally applied by direct coating methods such as low-temperature chemical vapor deposition, but the high reactivity of K metal causes side reactions during pretreatment and limits these techniques. To eliminate the need for anode pretreatment, researchers have proposed strategies for transferring the SEI layer onto the metal anode during battery cycling. Notably, David Mitlin’s team used double-coated tape-cast processing to deposit AlF_3_ on both sides of a PP separator (AlF_3_@PP) [159]. On the anode side, AlF_3_ reacts in situ with K metal to form a graded SEI of KF, AlF_3_ and Al_2_O_3_ (Fig. 8k). Its gradient structure effectively inhibits dendrite growth and enhances interface stability. On the cathode side, the AlF_3_ layer reduces polarization and accelerates electrochemical kinetics. It is worth noting that this functional separator demonstrates excellent wettability regulation in ester-based electrolyte systems, with its ionic conductivity increasing by up to 32% (Fig. 8l). This provides an important reference for the compatibility design of different electrolyte systems. Based on this, the potassium-metal battery with K_3_[Fe(CN)_6_] cathode has a capacity retention rate of 91% after 100 cycles, demonstrating markedly better cycling stability than a conventional separator system. In Na–S systems, Wu’s group fabricated a Na_2_S/Na_2_Te@C heterostructured cathode that in situ generates Na_2_TeS_3_ interfacial layers (Fig. 8n) [160]. This interface reduces the Na^+^ diffusion barrier from 0.39 to 0.20 eV, accelerating desodiation kinetics (Fig. 8o, p), while the polytellurosulfides formed during cycling assemble into a dense SEI on the Na anode, suppressing dendrite growth for over 800 h in symmetric cells. Although the larger ionic radius of Na can exacerbate SEI instability, the robust covalent Te-S network in Na_2_TeS_3_ accommodates volume changes and sustains 363 mAh g^−1^ at a sulfur loading of 4.5 mg cm^−2^. The moderate electronegativity of tellurium compared to sulfur enhances electron distribution for stronger polysulfide adsorption and catalytic conversion, and the high Te-S bond energy (~ 250 kJ mol^−1^) imparts mechanical and chemical stability to the SEI. Together, LiTe_3_ and Na_2_TeS_3_ establish a dynamic balance between shuttle suppression and metal deposition stabilization. Future research should map structure–property relationships in tellurium compounds by tuning Te/S ratios and employ machine learning to screen multifunctional interfacial composites, aiming for ultrahigh sulfur utilization and extended cycle life.

Recent studies have revealed species-dependent polysulfide corrosion kinetics and inactive lithium evolution mechanisms at the alkali-metal anode, together with effective protection and mitigation strategies. In M-S batteries, uncontrolled side reactions between polysulfides and anodes severely compromise interfacial stability and cycling performance. The latest progress by Huang et al. demonstrates that high-order Li_2_S_8_/Li_2_S_6_ induces much faster corrosion than low-order Li_2_S_4_, while LiPS-derived SEI films provide limited protection [161]. Controlling the working window to favor low-order LiPS mitigates corrosion and improves cumulative capacity (~ 31%). Correspondingly, strategies such as WSEs, LiNO_3_ additives, and protective coatings/alloying have shown effectiveness in suppressing polysulfide-induced anode degradation. Moreover, continuous polysulfide corrosion accelerates inactive Li accumulation, which blocks ion transport and causes severe concentration polarization during discharge. This corrosion-induced heterogeneity increases Li^+^ diffusion impedance and accelerates capacity fading, while additives that reactivate inactive Li effectively delay anode failure and extend cycle life [162].

In addition to solid-state interface modifications, electrolyte-solvation regulation has recently emerged as a complementary strategy to mitigate polysulfide shuttle in alkali M-S batteries. Recently, WSEs have gained increasing attention due to their ability to construct heterogeneous solvation shells, thereby lowering polysulfide reactivity and mitigating cathode dissolution and anode corrosion. However, studies reveal that WSEs inherently slow sulfur redox kinetics: a “two-stage solvation mechanism” has been identified, where the direct coordination of weakly solvating solvents to polysulfides sharply increases activation polarization and retards interfacial charge transfer [163]. Furthermore, under lean-electrolyte conditions, WSEs induce a transition from 3D diffusion-controlled Li_2_S nucleation to 2D surface-controlled deposition, which reduces sulfur utilization [164]. To address these challenges, recent strategies combine WSEs with redox mediators, LiFSI-based formulations, or catalytic interlayers to enhance interfacial charge transfer and accelerate polysulfide conversion [165]. By integrating WSEs with interfacial engineering, both polysulfide solubility and reaction kinetics can be simultaneously regulated, paving the way for high-energy–density designs.

Despite these advances, liquid-electrolyte-based strategies still face intrinsic limitations in fully blocking polysulfide migration and dendrite formation, which motivates the development of SSEs.

### Interfacial Regulation of (Quasi-) Solid-State Electrolytes for Shuttle-Free M-S Batteries

To build on the liquid‐phase shuttle suppression strategies discussed earlier, researchers have turned to solid‐phase conversion by using quasi‐solid or fully solid electrolytes. These electrolytes reshape sulfur redox pathways by bypassing the formation of soluble high‐order M_2_S_n_ (4 ≤ n ≤ 8) and thus suppress the shuttle effect at its source. Accordingly, the development of (quasi-) SSEs tailored for Li/Na/K–S systems has emerged as a pivotal route to suppress the shuttle effect and enhance battery performance.

**Fig. 9:**
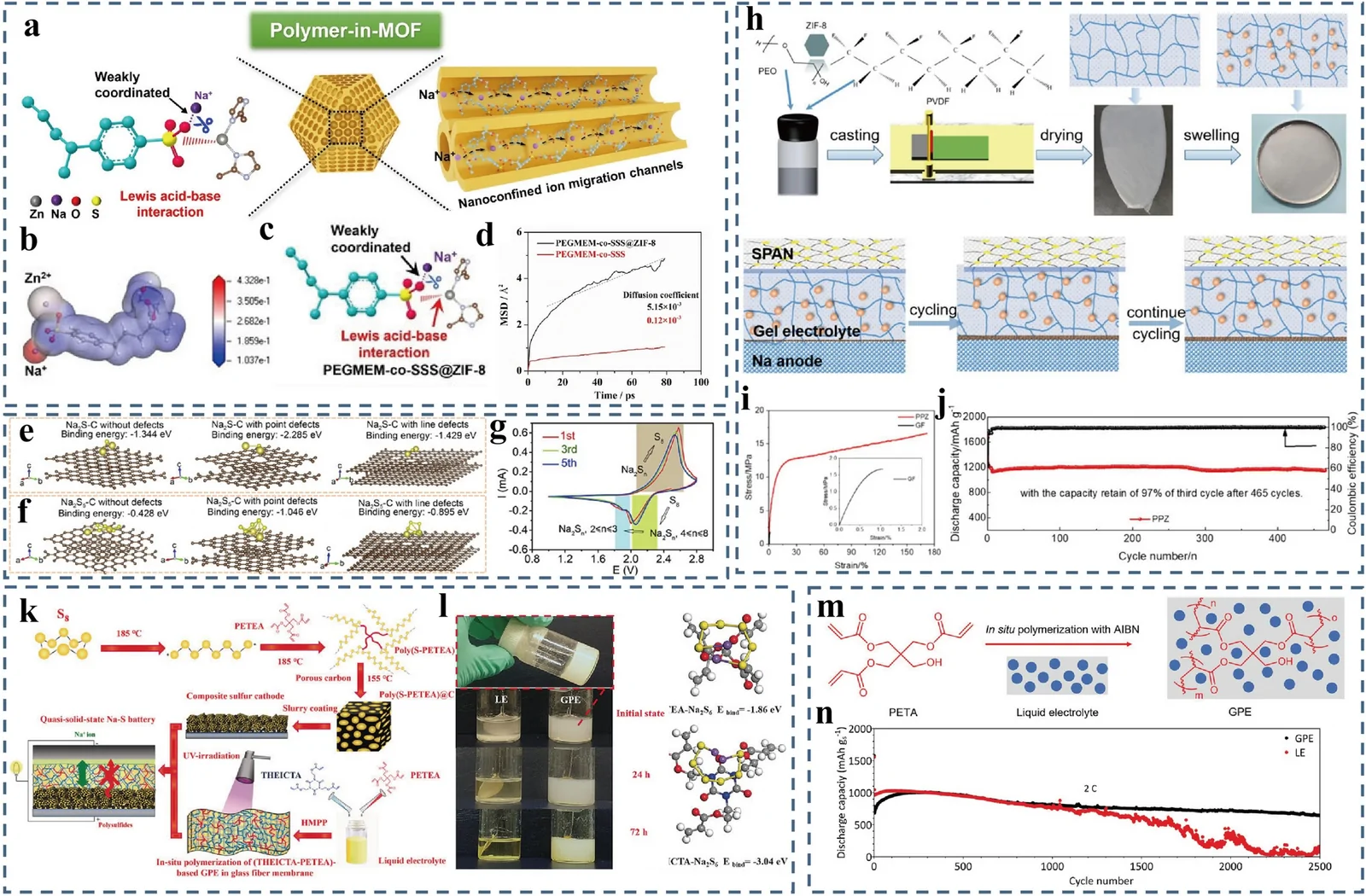
**a** Synthesis of the polymer-in MOF SICSPEs. **b** Electrostatic potential maps of the PEGMEM-co-SSS@ZIF-8. **c** Schematic illustration of the Lewis acid–base interactions in enhancing the Na^+^ dissociation. **d** The mean square displacement of Na^+^ ions in different SSEs. Reproduced with permission [167]. Copyright 2023, Wiley–VCH. **e, f** The stable models of Na_2_S and Na_2_S_5_ molecule adsorption on the carbon surface with or without different defects. **g** CV curves. Reproduced under terms of the CC-BY license [176]. Copyright 2020, The Authors, published by Elsevier Inc.** h** Schematic illustration of the designed gel electrolyte for Na anode. **i** Stress–strain curves of the PPZ membrane and GF separator. **j** Long cycling performance. Reproduced with permission [177]. Copyright 2024, Elsevier. **k** Preparation of the quasi-SSE Na–S battery. **l** Visual test (left). Calculated binding energies of Na_2_S_6_ with PETEA and THEICTA monomers (right). Reproduced with permission [11]. Copyright 2018, Wiley–VCH. **m** Synthesis of the GPE. **n** Long cycling performance. Reproduced with permission [179]. Copyright 2022, Wiley–VCH

Solid polymer electrolytes (SPEs) combine mechanical flexibility with excellent electrode compatibility, making them promising for mitigating the shuttle effect in M-S batteries. Polymer-inorganic composites and interface engineering have improved ionic conductivity and cycling life, but the larger Na^+^ and K^+^ radii still yield lower transference numbers and transport efficiency than Li^+^, worsening shuttle behavior in quasi-solid systems [166]. To address the critical challenges in solid-state Na metal batteries, Zhang and co-workers developed a "polymer-in-MOF" single-ion conducting electrolyte (PEGMEM-co-SSS@ZIF-8) [167]. This innovative design leverages the nanopores (1.1 nm) of ZIF-8 to spatially confine sulfonic acid group-containing copolymers (Fig. 9a). Strong Zn^2+^-SO_3_^−^ interactions (-12.41 eV) boost Na^+^ dissociation, yielding a transference number of 0.87 and conductivity of 4.01 × 10^–4^ S cm^−1^ at 80 °C (Fig. 9b, c). Molecular dynamics calculation shows a 43-fold increase in Na^+^ diffusion under confinement (Fig. 9d). In Na_3_V_2_(PO_4_)_3_/Na cells, this electrolyte retains 96% capacity over 300 cycles at 1 C, and symmetric Na||Na cells operate dendrite-free for 600 h, demonstrating its superior interfacial stability and dendrite suppression.

Gel polymer electrolytes (GPEs) exhibit high ionic conductivity by inducing crystalline microregions in liquid electrolytes (LE) and forming a 3D network structure through hydrogen bonds or chemical cross-linking. This structure retains the rapid cation (such as Li^+^, Na^+^, K^+^) transport channels similar to those of LE while also possessing the mechanical stability of solids. For M-S batteries, an ideal GPE should combine: (1) polar functional groups to solvate the metal salt; (2) excellent mechanical, chemical, and thermal stability; (3) low crystallinity and a suitable glass transition temperature to facilitate rapid ion migration; (4) room‐temperature conductivities > 10^–3^ S cm^−1^ and a wide electrochemical stability window; (5) sufficient flexibility and mechanical robustness to accommodate electrode volume changes and suppress dendrite formation. Common polymer matrices for GPEs include PEO, PVDF‐HFP, PVDF, and PMMA, etc. [168–171].

PVDF combines low cost with excellent compatibility in M-S batteries. Its micropores promote rapid ion transport while partially restricting polysulfide diffusion, thus enhancing cycle stability. Early work by Ahn et al. employed PVDF-based gels in Li/Na–S batteries, achieving an initial 489 mAh g^−1^ in Na–S cells but suffering a sharp drop to 40 mAh g^−1^ after 20 cycles [172, 173]. PVDF-HFP copolymers exhibit lower crystallinity, a wider electrochemical window, and superior flexibility, making them a more optimal choice [174]. However, the gel alone cannot anchor polysulfides effectively, so inorganic fillers such as SiO_2_ and TiO_2_ are added to both fill pores and maintain continuous ion channels [175]. For example, Li and Chen combined a PVDF-HFP/SiO_2_ gel with defective nano-carbon to suppress shuttle effects and accelerate sulfur conversion at the cathode/gel interface, markedly improving bulk cathode performance (Fig. 9e-g) [176]. Zhu et al. fabricated a composite gel by blending hydroxypropyl cellulose, PVDF-HFP, and SiO_2_, achieving enhanced ionic conductivity. Moreover, porous gels based on PEO-NaFSI-TiO_2_ and PVDF-HFP-TiO_2_ reached conductivities of 4.89 × 10^–4^ S cm^−1^ (high temperature) and 1.3 × 10^–3^ S cm^−1^ (at room temperature), respectively [175]. However, both systems suffered from interfacial passivation and polysulfide shuttle, which limited their capacity retention. Introducing ZIF-8 nanoparticles into PVDF/PEO blend gels reduces polymer crystallinity, optimizes microporosity and cation-framework interactions (Fig. 9h) [177]. The resulting dense matrix regulates Na^+^ flux and inhibits dendrite growth (Fig. 9i, j). Thanks to the flexibility of the blend polymer (tensile strength 16.5 MPa, elongation 171%), the CEI and SEI have good stability, enabling the RT Na–S battery to achieve a high discharge specific capacity and long cycle performance of 1585.4 mAh g^−1^. Co-filling PVDF-HFP films with Lewis-acidic additives like boron nitride or metal–organic complexes enables selective polysulfide adsorption and ion flux control, achieving 92% capacity retention over 2000 cycles at 5 C in dual-ion cells [178]. Sundara et al. introduced boron nitride into a PVDF-HFP/poly(butyl methacrylate) membrane for the first time in RT Na–S cells, demonstrating that the electronegative N and B sites efficiently capture NaPS and substantially enhance both safety and cycling performance [77]. In summary, gel electrolytes provide flexible frameworks and excellent interfacial contact. However, careful polymer-inorganic composite design is vital to achieve high ionic conductivity, strong polysulfide adsorption, and mechanical/chemical robustness, thereby alleviating shuttle effects and interface by-product accumulation.

Currently, most GPEs rely on ex-situ techniques such as solvent casting (heterogeneous) and phase inversion (homogeneous) for synthesis. These multi-step processes are complex, which restricts their promotion in commercial energy storage. Wang Guoxiu’s team took the lead in proposing the idea of in-situ synthesis and successfully prepared the cathode and gel electrolyte based on poly(S-PETEA)@C through monomer copolymerization (Fig. 9k) [11]. This GPE chemically anchors sulfur species to suppress the shuttle effect, delivers higher Na^+^ transference numbers and interfacial stability than traditional gels. To visually characterize the formation and diffusion of NaPS in the electrolyte, equal amounts of sulfur powder and Na foil were immersed in LE or GPE to observe the color change of the electrolyte. No obvious change occurred in GPE, while the color of LE became darker. DFT calculations show THEICTA groups bind Na_2_S_6_ with -3.04 eV energy, far surpassing PC and FEC, indicating that NaPS is more likely to be infixed rather than diffused (Fig. 9l). Buchmeiser’s team then prepared a GPE for RT Na–S batteries via in situ free radical polymerization of PETA and LE using AIBN as initiator (Fig. 9m) [179]. Because polymerization occurs directly on the Na anode and sulfur cathode surfaces, the GPE forms intimate electrode contacts and delivers an ionic conductivity of 2.33 mS cm^−1^ at 25 °C. Its cross-linked network immobilizes solvent molecules and encapsulates the cathode, providing polysulfide adsorption sites that suppress shuttling and promote faster conversion kinetics. As a result, the Na–SPAN full cell retains over 600 mAh g^−1^ after 2500 cycles at 2 C (Fig. 9n). By immobilizing electrolyte within a polymer matrix and eliminating free solvent while preserving liquid-like ion transport, in-situ polymerized GPEs reduce side reactions and markedly boost cycling stability, safety, and overall battery performance.

**Fig. 10:**
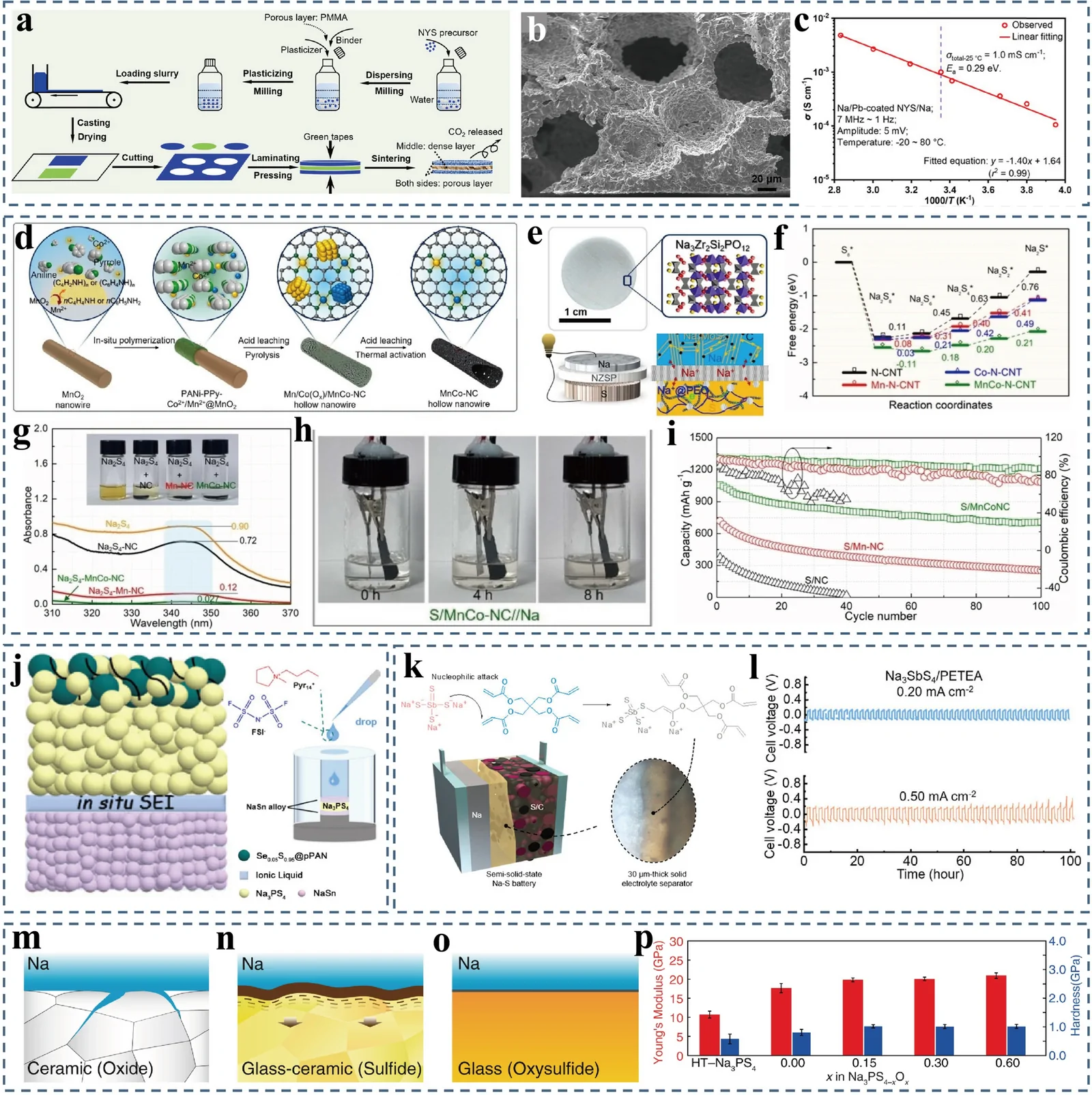
**a** Preparation process of NYS trilayers. **b** SEM images. **c** Arrhenius plot of the temperature‐dependent conductivities in the Na||Na symmetric cell. Reproduced under terms of the CC-BY license [181]. Copyright 2023, The Authors, published by Wiley. **d** Synthesis process of MnCo-NC sulfur matrix. **e** Photograph and crystal structure of the NZSP. **f** Gibbs free energy of sulfur reduction process on different matrixes. **g** Visualized adsorption tests and the UV/Vis spectrums. **h** Photographs of vial cells with S/MnCo-NC cathodes. **i** Long-cycling performance. Reproduced with permission [182]. Copyright 2023, Wiley–VCH. Schematic diagram of **j** NaSn/Na_3_PS_4_/NaSn batteries; Reproduced with permission [185]. Copyright 2020, American Chemical Society. **k** Na_3_SbS_4_/PETEA hybrid electrolyte. **l** Cyclability of symmetric cell. Reproduced with permission [186]. Copyright 2020, American Chemical Society. **m–o** Classification of SSE-Na metal interfaces. **p** Young’s modulus and hardness of various SSEs. Reproduced under terms of the CC-BY license [90]. Copyright 2022, The Authors, published by Springer Nature

Inorganic SSEs offer high ionic conductivity, mechanical stability and non-flammability, but their brittleness and poor electrode contact limit practical use. To address this, one study infiltrated ceramic SSE films with PVDF-HFP, creating a “polymer-in-ceramic” sodium conductor that also suppressed the shuttle effect in RT Na–S batteries [180]. In addition, Yang et al. developed a self-supporting Na_3_YSi_4_O_12_ (NYS) ceramic scaffold with a porous/dense/porous trilayer structure via a tape-casting technique, offering an innovative solution for pressureless all-solid-state Na–S batteries (Fig. 10a) [181]. Among them, a 90 μm dense core acts as a transport membrane for Na^+^ and 170 μm porous outer layers (68% porosity) to host the sulfur cathode and buffer volume changes while maintaining ion pathways (Fig. 10b). The assembled Na/NYS/S full cell demonstrated a high initial discharge capacity of 970 mAh g^−1^ and remarkable cycling stability (62% capacity retention after 150 cycles) under pressure-free conditions. Meanwhile, Na||Na symmetrical cells ran dendrite-free for 460 h. The NYS scaffold achieves 1.0 mS cm^−1^ conductivity, 0.29 eV activation energy and an > 8 V stability window (Fig. 10c). By combining polymer infiltration and tailored ceramic architecture, these works pave the way for scalable, high-energy–density all-solid-state M-S batteries. Moreover, embedding inorganic nanoparticles (such as LLZO, LAGP, etc.) into polymer matrices (such as PEO, PVDF, etc.) also significantly enhances their ionic conductivity and mechanical properties. Recently, Zhang et al. reported a hollow carbon nanotube supported MnN_4_/CoN_4_ diatomic catalyst (MnCo-NC) via a sacrificial template process that lowers the Na_2_S decomposition barrier from 1.56 to 0.62 eV (on a pure carbon substrate) and chemisorbs polysulfides with a binding energy of -2.15 eV (Fig. 10d, e) [182]. When integrated with a Na_3_Zr_2_Si_2_PO_12_/PEO-NaFSI electrolyte to form a 3D ion conduction network, this system enables all-solid-state Na–S batteries to cycle with 67% capacity retention over 100 cycles at 1.5 g cm^−3^ sulfur loading and an average coulombic efficiency of 98.5%. By synergistically optimizing atomic-scale catalytic site design and SSE interface engineering, this work provides a groundbreaking approach for developing low-temperature all-solid-state M-S batteries (Fig. 10f-i). The achieved performance, notably an energy density of 1008 Wh kg^−1^, comparable to LE systems, marks a critical step toward practical applications in this field.

Sulfide-based SSEs have attracted extensive attention due to their excellent compatibility with sulfur cathodes, high ionic conductivity at room temperature, low grain boundary resistance, and good formability. However, its electrochemical stability window is narrow and it is prone to decomposition when in direct contact with active metals (Li, Na, K). For example, Na_3_PS_4_ reacts with Na metal to form Na_2_S and Na_3_P, disrupting ion transport, raising polarization and accelerating performance decay [183, 184]. To stabilize this interface, An et al. used a NaSn alloy anode and introduced the ionic liquid Pyr_14_FSI into the electrolyte (Fig. 10j), which in situ generates a robust SEI on the metal surface during cycling, and simultaneously doped 5 mol% selenium into S@pPAN cathodes to boost electronic conductivity and restrain polysulfide loss [185]. In addition, to address the issues of parasitic reactions and mechanical brittleness in sulfide-based SSEs (e.g., Na_3_SbS_4_), Ren et al. proposed an innovative inorganic/organic hybrid SSE [186]. By implementing an in situ cross-linking reaction between Na_3_SbS_4_ and PETEA polymer, a flexible and transferable hybrid electrolyte film was fabricated, resulting in a 30 µm hybrid film that has an areal resistance of just 65 Ω cm^2^, which can suppress both the dissolution of Na_3_SbS_4_ and the interfacial side reactions (Fig. 10k, l). Covalent bonds formed during crosslinking electronically insulated the SSE and reduced Na_3_SbS_4_ solubility in LE by 90% (confirmed via UV–vis), stabilizing the interface with Na metal. Chi et al. synthesized fully dense oxysulfide glasses (Na_3_PS_4−x_O_x_, 0 < x ≤ 0.6) via mechanochemical milling, achieving a Young’s modulus of 20.9 GPa and hardness of 1.0 GPa [90]. The incorporation of bridging oxygen units reinforced the glass network, effectively suppressing Na dendrite penetration and enabling symmetric cells to achieve a critical current density (CCD) of 2.3 mA cm^−2^ at 60 °C (Fig. 10m-p). In a trilayer Na_3_PS_3.4_O_0.6_|Na_3_PS_3.85_O_0.15_|Na_3_PS_3.4_O_0.6_ architecture, these oxysulfides deliver 1280 mAh g^−1^ at 0.1 mA cm^−2^ with over 80% capacity retention after 40 cycles. Together, SEI engineering, polymer-inorganic hybrids and oxygen-doped glass electrolytes offer scalable, high‐performance solutions for stable sulfide interfaces in Na–S batteries.

**Fig. 11:**
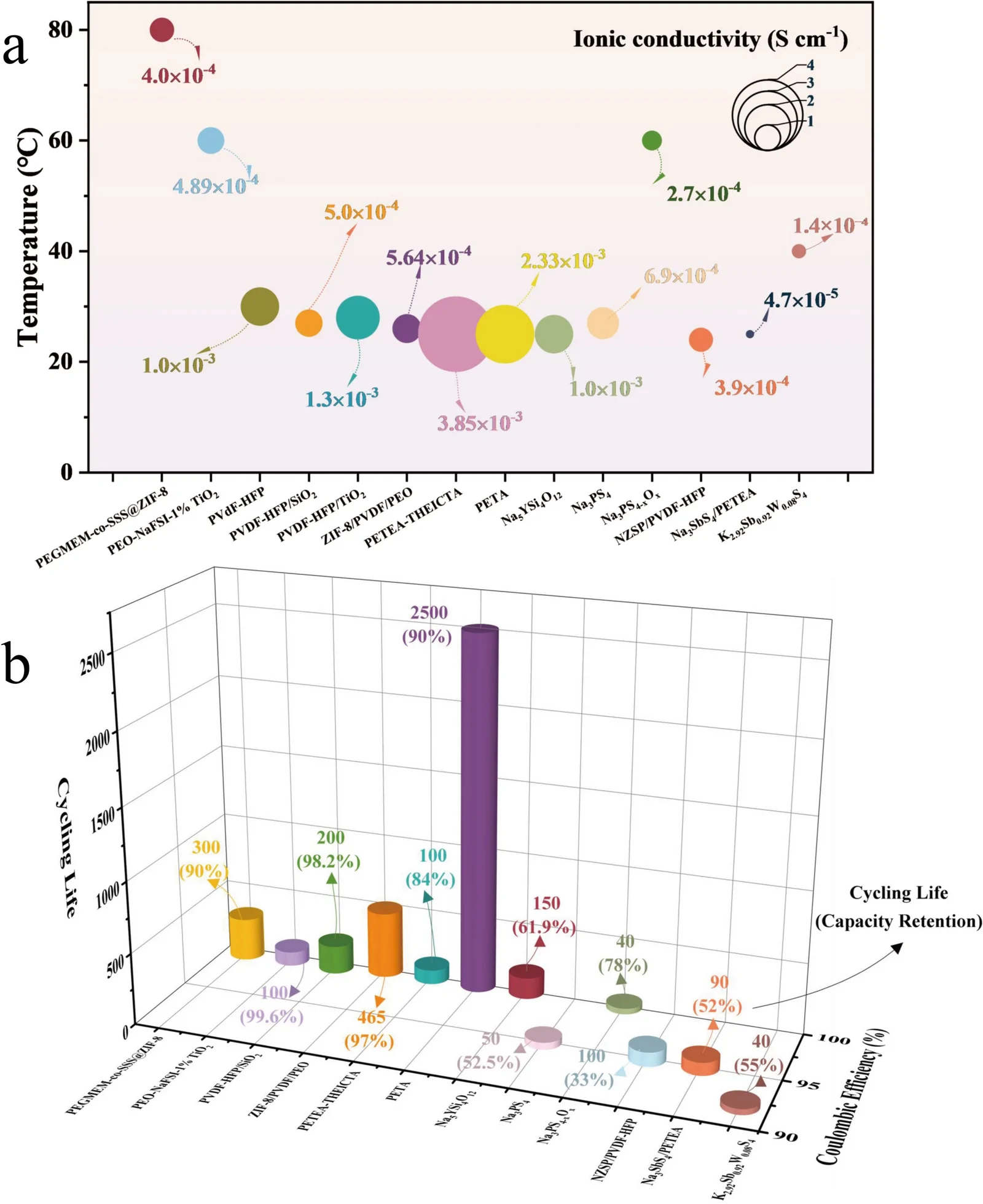
**a** Ionic conductivity of SSE materials for M-S batteries at different temperatures. **b** Specific capacity retention and coulombic efficiency of the SSEs materials for M-S batteries at different cycling lives

In contrast, the development of inorganic SSEs for K–S batteries is still at an early stage due to the larger K^+^ ionic radius, lower solvation energy, and slower reaction kinetics compared with Na^+^ and Li^+^. Nevertheless, several studies have reported achieved notable breakthroughs that provide promising pathways toward high-energy–density and stable K–S systems. Lu et al. prepared a K-β”-Al_2_O_3_ solid electrolyte, demonstrating excellent interfacial wettability with molten potassium and achieving an initial discharge capacity of ≈402 mAh g^−1^ at 150 °C, along with stable cycling over 1000 cycles [187]. A K_2_Fe_4_O_7_-based superionic conductor exhibited a high ionic conductivity of ≈2.2 × 10^–3^ S cm^−1^ at RT and improved interfacial compatibility with K metal anodes. Its integration enabled faster K^+^ transport, lower polarization, and better cycling stability, showing strong potential for scalable and cost-effective SSE applications [188]. More recently, Shao et al. introduced K_3_SbS_4_ as a potassium superionic conductor with the low activation energy of 0.27 eV and the ionic conductivity of ≈2.5 × 10^–6^ S cm^−1^ (RT), which was further improved to 7.7 × 10^–5^ S cm^−1^ through W doping (K_2.92_Sb_0.92_W_0.08_S_4_) [189]. The doped electrolyte also showed strong chemical stability against KPS, effectively inhibiting the shuttle effect. Collectively, these advances highlight the importance of optimizing crystal structures, vacancy engineering, and interfacial chemistry to develop high-conductivity, dendrite-resistant SSEs, offering a promising pathway toward practical all-solid-state K–S batteries (Fig. 11).

Overall, SSEs have emerged as a pivotal research direction in alkali M-S batteries due to their potential to enhance safety, suppress dendrite growth, and inhibit polysulfide shuttling. However, poor interfacial contact with electrodes often increases impedance and restricts ion transport, limiting overall performance. To overcome these issues, each shows distinct advantages and limitations across Li/Na/K–S systems. Inorganic SSEs deliver high ionic conductivity (> 10^–3^ S cm^−1^) and strong mechanical rigidity, which help suppress dendrite formation and polysulfide shuttle. However, their brittleness and poor interfacial compatibility hinder performance, particularly in Na–S and K–S batteries. SPEs provide excellent electrode–electrolyte contact and flexibility, reducing interfacial resistance, yet their low conductivity limits use under high-loading/rate conditions. Inorganic/organic hybrid SSEs integrate the benefits of both, embedding ceramic fillers into polymer matrices to enhance ion conduction, interfacial stability, and mechanical strength. As a result, inorganic/organic hybrid SSEs achieve improved sulfur utilization, better polysulfide suppression, and enhanced cycling performance. The critical challenge is to engineer SSE/electrode interfaces that combine excellent compatibility with multifunctional synergies, enabling efficient, safe, and long-cycling M-S batteries.

## Conclusion and Perspective

### Conclusion

Alkali M-S batteries are regarded as strong candidates for the next-generation energy storage systems due to their high theoretical energy density and potential low cost. However, these systems face critical problems such as polysulfide shuttle, slow redox kinetics of sulfur, and dendrite growth of the metal anode. In recent years, interface engineering has become a promising strategy to mitigate the shuttle effect in M-S batteries. There are various strategies, spanning from the design of porous cathodes, construction of artificial interfacial films on anodes, to the functionalization of separators and the development of (quasi-) SSEs. This review focuses on discussing multiple strategies of interface engineering in alkali M-S batteries for inhibiting polysulfide shuttling, promoting sulfur redox reactions, and stabilizing metal anodes. By ion-selective mechanisms, chemical adsorption, and catalytic conversion, researchers have successfully reduced the migration of polysulfides and enhanced the cycle stability and coulombic efficiency of batteries.

Because Na^+^ and K^+^ ions have larger ionic radii, they exhibit weaker solvation and stronger electrostatic associations with polysulfide anions, resulting in a lower apparent solubility of NaPS/KPS in ether-based electrolytes compared with LiPS. Consequently, one might expect stronger suppression of the shuttle effect. However, Na–S and K–S batteries often experience more rapid performance decay, indicating that additional interfacial phenomena dominate their failure mechanisms. For this result, we propose the following explanation. Despite broadly similar interface-engineering strategies to those employed in Li–S systems, Na/K polysulfides exhibit distinct solution chemistry. Stronger cation-polysulfide binding induces the formation of compact ion-polysulfide aggregates or even gel-like networks, which hinder ion transport and slow sulfur conversion kinetics. In parallel, the weaker solvation of Na^+^/K^+^ exacerbates uneven alkali-metal deposition and interfacial passivation at the anode, further accelerating capacity degradation. These mechanistic insights primarily apply to ether-based electrolytes, where the relatively high donor numbers enhance polysulfide solvation for Li species, while NaPS/KPS remain only partially soluble yet still prone to aggregate-driven transport limitations. By contrast, carbonate-based Na–S systems represent an important exception: their intrinsically low donor numbers and strong polarity substantially suppress polysulfide dissolution, thereby partially mitigating conventional shuttle effects [20]. Nevertheless, the thermodynamic incompatibility between carbonate solvents and NaPS induces nucleophilic solvent decomposition and the formation of insulating interfacial byproducts (e.g., Na_2_CO_3_, Na_2_SO_3_, Na_2_SO_4_), ultimately leading to electrode passivation and electrolyte depletion. As a result, carbonate-based Na–S batteries fail via a fundamentally different degradation pathway compared with ether-based systems [24].

In past studies, Li–S and Na–S batteries have successfully achieved excellent electrochemical performance through various functional modification strategies, but mature strategies have not been directly applied to K–S batteries. For instance, Tanibata et al. prepared a Na_3_PS_4_ glass–ceramic electrolyte with high ionic conductivity based on Li_3_PS_4_ sulfide glass–ceramics by completely replacing Li^+^ with Na^+^ [190]. A high discharge capacity of approximately 1100 mAh g^−1^ and excellent cycling stability were achieved in the RT all-solid-state Na–S battery. However, no literature reports on the use of K_3_PS_4_ in K–S batteries have been seen so far. On the one hand, this might be due to various limitations, such as the large radius of K^+^ and the preparation process. On the other hand, if these bottlenecks are addressed through doping modification, framework design or composite strategies, some not-yet-studied materials may bring new research directions to K–S batteries in the future.

### Perspective

Based on the above, while interfacial engineering strategies for Li/Na/K–S batteries share common themes, such as polar adsorption, catalytic interlayers, artificial SEI formation, and the use of solid/gel electrolytes, they must be tailored to the specific ionic radii, polarization strengths, and polysulfide solubilities of each system. Future efforts should concentrate on several key directions:

(a) *Dynamic, self-adaptive interfaces.* Utilizing reversible phase change materials or self-healing polymer networks, the interface components can spontaneously adjust their structure and chemical stability during charge/discharge processes to cope with the interface degradation caused by continuous cycling. In view of the large interfacial stresses caused by repeated sulfur conversion and volume changes, designing a phase-tunable hybrid interfacial network combining ion-conductive polymers and reversible inorganic frameworks is a very promising direction. For example, by embedding Li/Na/K-conductive phase-change domains (e.g., Na_3_SbS_4_, K_3_SbS_4_, LLZO nanodomains) into dynamic covalent polymer matrices, the interfacial layer could self-reorganize under electrochemical stress to maintain low impedance and suppress microcrack formation. Such hybrid architectures could provide a pathway toward self-healing, shuttle-resistant interfaces.

(b) *Cross-scale interface control and integration*. Combine atomic-scale chemical modifications, mesoporous scaffold architectures, and macroscopic coating techniques to build a hierarchical interface network that remains stable from the nanoscale through to the full electrode. At the atomic scale, heteroatom doping and single-atom catalysts can modulate local electronic structures and lower M_2_S nucleation barriers to accelerate redox kinetics. At the mesoporous scale, MXene, MOF/COF, and hollow carbon frameworks offer interconnected ion channels and abundant active sites, facilitating fast ion transport and effective polysulfide confinement. At the macroscopic level, adaptive polymer coatings and hydrogel membranes buffer volume changes and stabilize electrode–electrolyte interfaces under high sulfur loading.

(c) *Industrially scalable interface innovations*. Develop cost-effective, process-friendly methods, such as continuous roll-to-roll coating or dry powder deposition, that can be readily upscaled, ensuring uniform interfacial construction and reproducible performance at the battery-manufacturing level. Besides, water-processable polymer binders, biomass-derived carbon coatings, and solution-synthesized catalytic interlayers could minimize processing costs while maintaining functionality.

(d) *AI‑Driven Predictive Interface Design.* Leverage machine-learning and big-data frameworks, similar to “genetic” algorithmic design, to pre-screen and optimize interfacial materials for M-S systems. By encoding descriptors such as ionic radius, binding energy, and solubility parameters into trained models, one can rapidly generate candidate chemistries and architectures, prioritize the most promising formulations, and guide targeted synthesis and testing. This data‑driven approach will accelerate the discovery of multifunctional coatings, catalysts, and electrolytes tailored to suppress shuttle effects across all alkali M-S battery interfaces.

Interfacial engineering strategies to suppress the polysulfide shuttle have progressed from passive containment to active, tunable approaches. Future advancements will critically depend on integrating interdisciplinary expertise from materials science, electrochemistry, and engineering. Insights derived from Li–S research provide invaluable guidance for Na–S and K–S systems. For instance, established Li–S electrocatalytic interface designs can be adapted, and first-principles calculations employed to screen optimal material combinations. However, inherent differences in the properties of Li⁺, Na⁺, and K⁺ ions necessitate system-specific solutions. By leveraging multi-dimensional interface architectures, dynamic response mechanisms, cross-scale optimization, and AI-driven predictive design, Li/Na/K–S batteries are poised to achieve transformative improvements in energy density, cycling stability, and safety. This progress will accelerate the commercialization of cost-effective, high-performance energy storage technologies.
